# Supplementary material for: Start-Stop Assembly: a functionally scarless DNA assembly system optimized for metabolic engineering
Source: Nucleic Acids Res. 2018 Nov 20;47(3):e17. doi: 10.1093/nar/gky1182 (PMC6379671; doi:10.1093/nar/gky1182)

Note S1. Start-Stop Assembly Quick-Start Guide

Start-Stop Assembly: a functionally scarless DNA assembly framework optimised for metabolic engineering.

George M. Taylor, Paweł M. Mordaka and John T. Heap*
Imperial College Centre for Synthetic Biology, Department of Life Sciences, Imperial College London, London, SW7 2AZ, United Kingdom.

Prepare genetic parts and vectors:

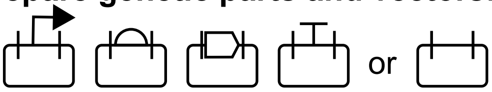

Review Start-Stop Assembly core vectors:

Table 1

if new vector needed →

How to make alternative destination vectors:

Figure S6

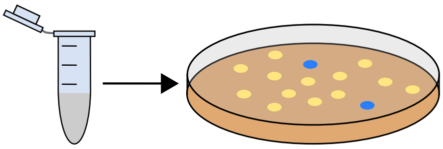

Review parts stored in pStA0:

Table S3

→

To construct and store new parts:

Note S2 | Table S1 | Table S2 | Table S8 | Figure S3

Plan assemblies:

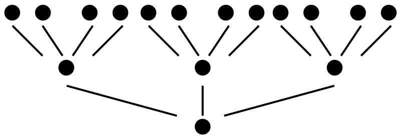

For choice of Start-Stop Assembly vectors:

Table 1 | Figure 4

If spacers required:

Table S5

Generic examples:

Figure S12-14

Specific examples:

Figure S16-20

Perform Start-Stop Assembly reactions:

Note S3
Lab Protocol

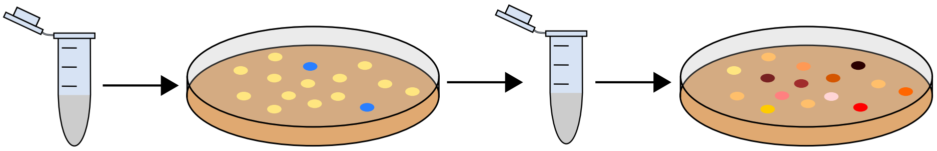

Supplement: Supplementary Data [file gky1182_supplemental_files.zip › NoteS1_Quick_User_Guide.pdf]
