# Supplementary material for: Start-Stop Assembly: a functionally scarless DNA assembly system optimized for metabolic engineering
Source: Nucleic Acids Res. 2018 Nov 20;47(3):e17. doi: 10.1093/nar/gky1182 (PMC6379671; doi:10.1093/nar/gky1182)
Supplement: Supplementary Data [file gky1182_supplemental_files.zip › NoteS3_Lab_Protocol.pdf]

Note S3. Start-Stop Assembly Lab Protocol

Start-Stop Assembly: a functionally scarless DNA assembly framework optimised for metabolic engineering.

George M. Taylor, Paweł M. Mordaka and John T. Heap*
Imperial College Centre for Synthetic Biology, Department of Life Sciences, Imperial College London, London, SW7 2AZ, United Kingdom.

Storing parts in Level 0

- Design primers for amplification of genetic part, or synthesize (refer to Note S2, Table S1 and S2 for prefix and suffix sequences and corresponding primer tails).
- PCR-amplify genetic parts (Figure S3), gel-purify PCR products.
- Level 0 Start-Stop Assembly reactions contained 20 fmol of empty vector pStA0, 40 fmol of insert (PCR product or synthetic DNA), T4 DNA Ligase buffer, 400 units of T4 DNA Ligase (typically use 1 µl) and 10 units of BsaI (typically 1 µl) in a total reaction volume of 20 µl.
- Reactions were incubated using a thermocycler for 30 two-step cycles of 37 °C for 5 minutes then 16 °C for 5 minutes, before a single final denaturation step at 65 °C for 20 minutes.
- Transform *E. coli* with reaction and plate onto LB agar plates containing ampicillin (100 µg ml^{-1}), IPTG (0.1 mM) and X-Gal (40 µg ml^{-1}).
- Pick single white colonies, sequence inserts using primers oligoGT234 and oligoGT235

Level 1 Start-Stop Assembly

- Refer to Figure 4 and Table 1 for choice of Level 1 vector.
- Level 1 Start-Stop Assembly reactions contained 20 fmol of Level 1 destination vector plasmid DNA, 40 fmol of each insert (plasmid DNA or annealed oligonucleotides), T4 DNA Ligase buffer, 400 units of T4 DNA Ligase and 10 units of SapI in a total reaction volume of 20 µl.
- Note. for combinatorial assemblies use an equimolar mix of the parts at a working concentration of 40 fmol.
- Reactions were incubated using a thermocycler for 30 two-step cycles of 37 °C for 5 minutes then 16 °C for 5 minutes, before a single final denaturation step at 65 °C for 20 minutes.
- Transform *E. coli* with reaction and plate onto LB agar plates containing tetracycline (10 µg ml^{-1}), IPTG (0.1 mM) and X-Gal (40 µg ml^{-1}).
- Pick single white colonies, miniprep overnight culture. Note. If doing a combinatorial assembly pick all white colonies from transformation plates and immediately resuspend in P1 buffer (Qiagen), miniprep.

Level 2 Start-Stop Assembly

- Refer to Figure 4 and Table 1 for choice of Level 2 vector.
- Level 2 Start-Stop Assembly reactions contained 20 fmol of Level 2 destination vector plasmid DNA, 40 fmol of each insert (plasmid DNA), T4 DNA Ligase buffer, 400 units of T4 DNA Ligase and 10 units of BsaI in a total reaction volume of 20 µl.
- Reactions were incubated using a thermocycler for 30 two-step cycles of 37 °C for 5 minutes then 16 °C for 5 minutes, before a single final denaturation step at 65 °C for 20 minutes.
- Transform *E. coli* with reaction and plate onto LB agar plates containing kanamycin (50 µg ml^{-1}), IPTG (0.1 mM) and X-Gal (40 µg ml^{-1}).
- Pick single colonies if final construct. Note. If doing a combinatorial assembly into Level 3 pick all non-blue colonies from transformation plates and immediately resuspend in P1 buffer (Qiagen), miniprep.

Level 3 Start-Stop Assembly

- Refer to Figure 4 and Table 1 for choice of Level 3 vector.
- Level 3 Start-Stop Assembly reactions contained 20 fmol of Level 3 destination vector plasmid DNA, 40 fmol of each insert (plasmid DNA), T4 DNA Ligase buffer, 400 units of T4 DNA Ligase and 10 units of BbsI in a total reaction volume of 20 µl.
- Reactions were incubated using a thermocycler for 30 two-step cycles of 37 °C for 5 minutes then 16 °C for 5 minutes, before a single final denaturation step at 65 °C for 20 minutes.
- Transform *E. coli* with reaction and plate onto LB agar plates containing chloramphenicol (25 µg ml^{-1}), IPTG (0.1 mM) and X-Gal (40 µg ml^{-1}).
