# Supplementary material for: Start-Stop Assembly: a functionally scarless DNA assembly system optimized for metabolic engineering
Source: Nucleic Acids Res. 2018 Nov 20;47(3):e17. doi: 10.1093/nar/gky1182 (PMC6379671; doi:10.1093/nar/gky1182)
Supplement: Supplementary Data [file gky1182_supplemental_files.zip › Supplementary_Materials.pdf]

## Supplementary Materials

# Start-Stop Assembly: a functionally scarless DNA assembly system optimised for metabolic engineering.

**George M. Taylor, Paweł M. Mordaka and John T. Heap***
Imperial College Centre for Synthetic Biology, Department of Life Sciences, Imperial College London, London, SW7 2AZ, United Kingdom.

*Email: j.heap@imperial.ac.uk

## SUPPLEMENTARY FIGURES

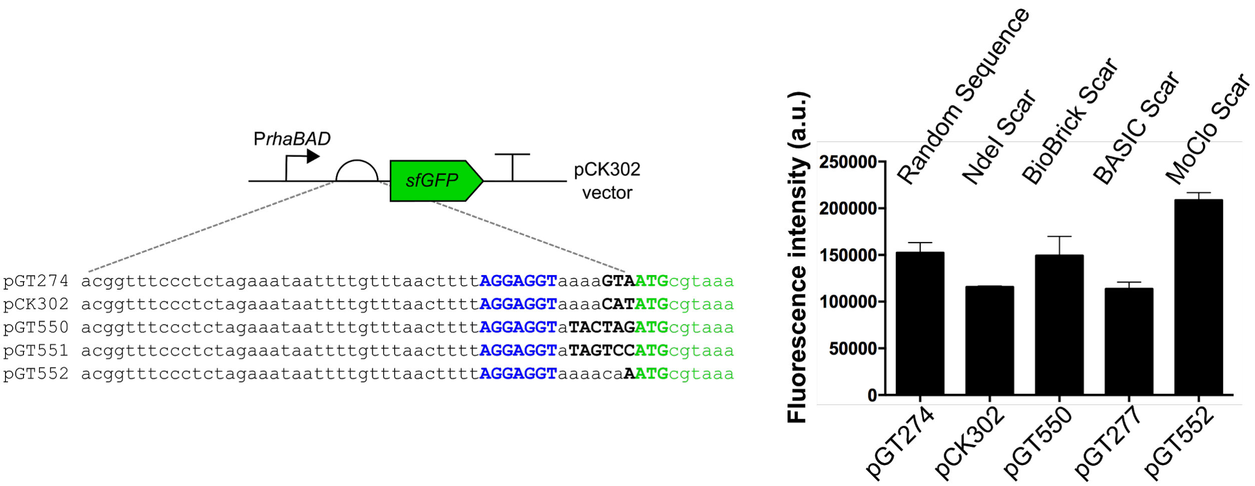


**Figure S1. Effect of DNA assembly scars immediately upstream of the start codon on expression of *sfGFP*.** The scar sequences (shown in **bold text**) that would result from NdeI-start codon cloning (pCK302), BioBrick assembly (pGT550), MoClo assembly (pGT552) or BASIC assembly (pGT277) as well as a random sequence (pGT274) were each incorporated into derivatives of the reporter plasmid pCK302 (1) between the Shine-Dalgarno sequence (blue sequence) of the RBS and the start codon (ATG) of *sfGFP* (green sequence) using inverse PCR. *E. coli* was independently transformed with each plasmid and transformants were characterised by flow cytometry. Fluorescence values presented represent the fluorescence of cells in mid--exponential phase of growth measured using flow cytometry with WT background subtracted. The error bars shown represent the standard deviation of three biological repeats. The results show that scars at this location have an impact on expression, but the impact of each scar depends on the context, including the CDS, so the impact of each scar will vary between different constructs.

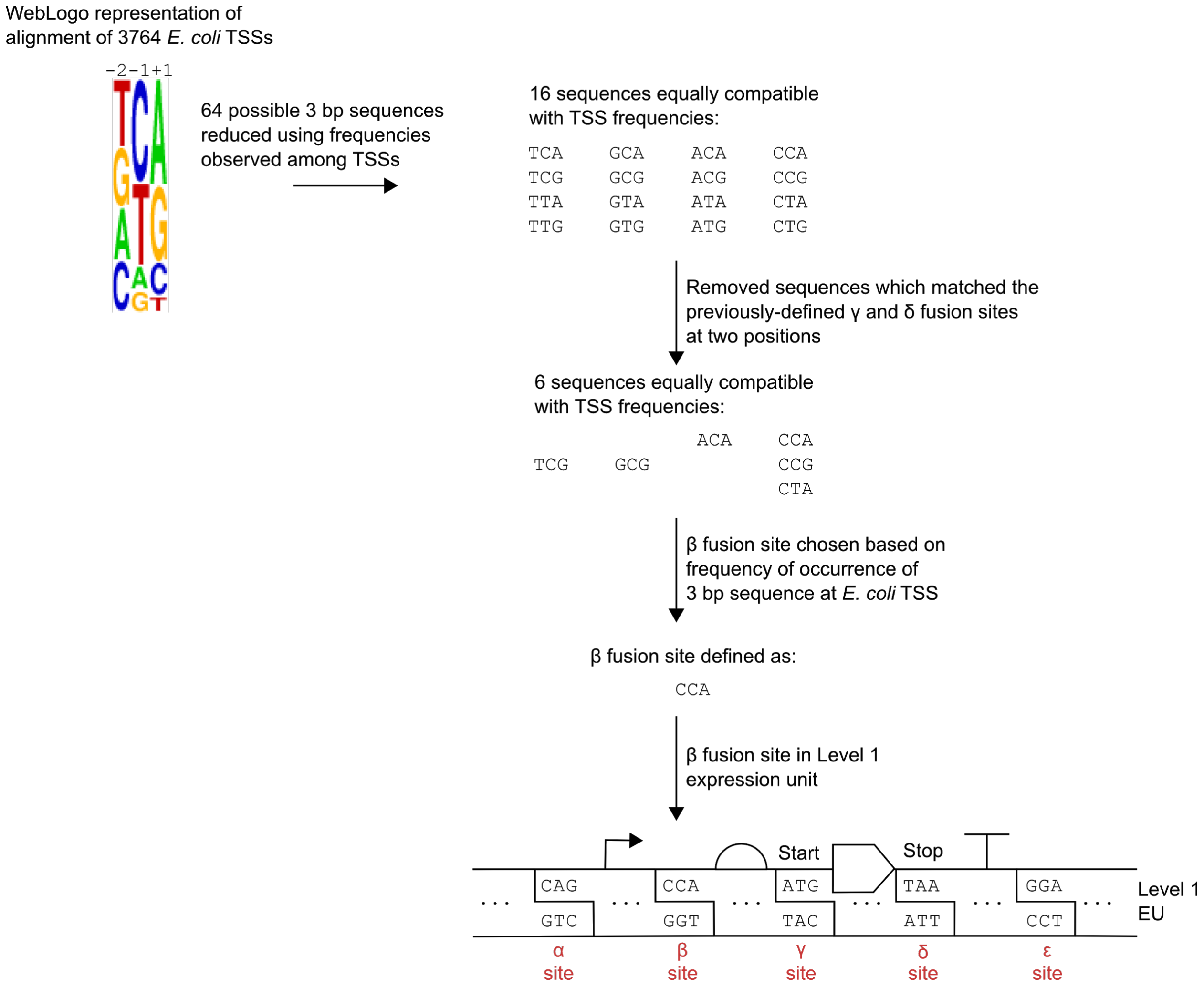


**Figure S2. Choice of β fusion site.** In an attempt to minimise the introduction of new sequences by the β fusion site at the junction between the promoter and the UTR/RBS, we tried to identify a consensus sequence for *E. coli* transcriptional start sites (TSS). We aligned 3746 previously-described *E. coli* MG1655 TSSs (2) which are visualised in the figure using WebLogo (3). The alignment showed a preference for A or G at the TSS +1 position, a preference for C or T at the -1 position, and no preference at the -2 position. First, we limited the candidate sequences for the β fusion site to the 16 sequences equally compatible with the observed preferences in the TSS. Next, to avoid misassembly we removed any of the candidate sequences which matched the already defined γ (ATG) and δ (TAA) fusion sites at two positions, which left six potential fusion sites. Finally, we compared the frequencies of these six candidate sequences among the 3746 previously-described *E. coli* MG1655 TSSs. The most frequently-occurring were ACA, which occurred 178 times, and CCA, which occurred 175 times. ACA is palindromic so would be a poor choice of fusion site, therefore we defined the β fusion site as CCA.

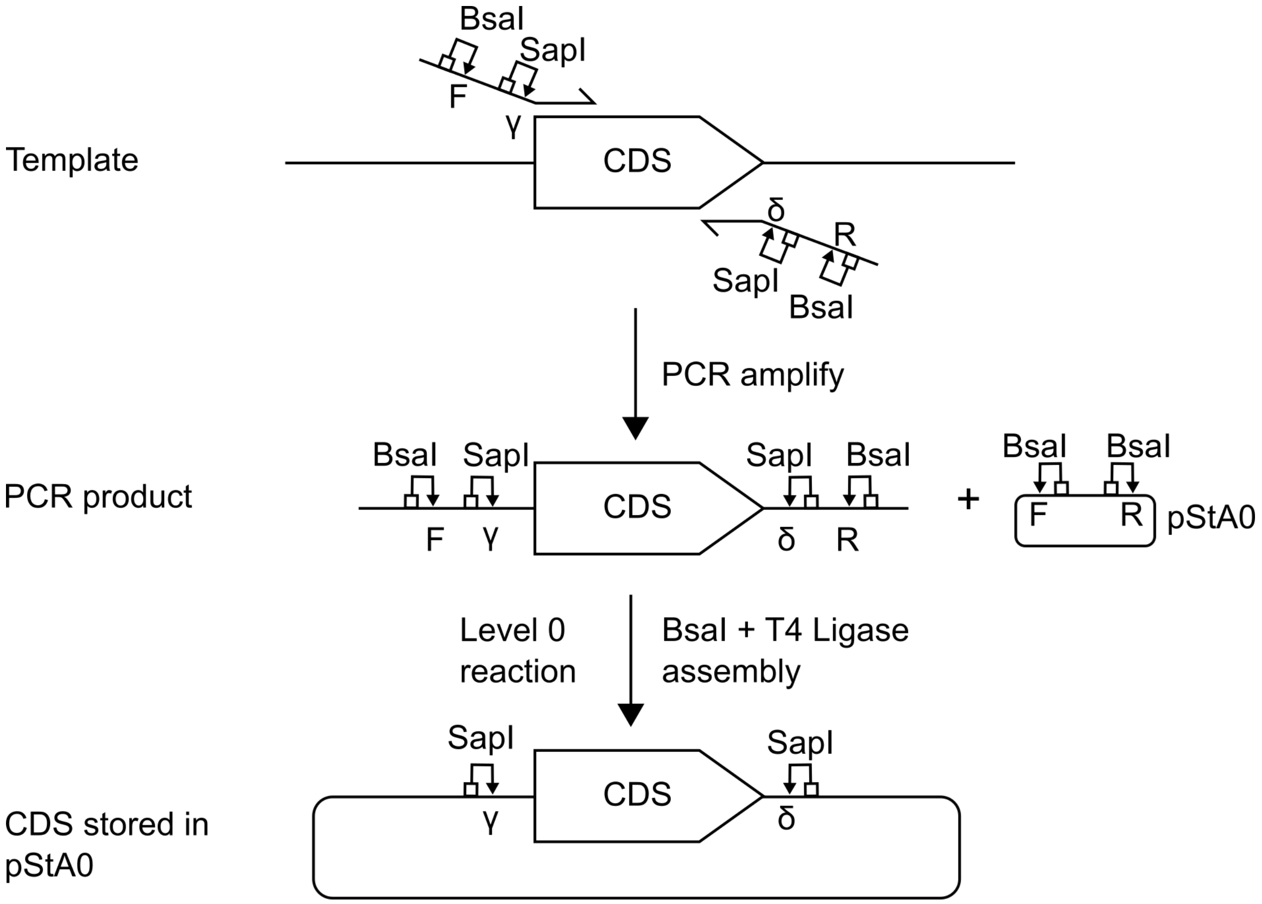


**Figure S3. Strategy for PCR-amplification and cloning of genetic parts into Level 0 vector pStA0.** To be used in Start-Stop Assembly, each genetic part must be flanked by the appropriate prefix and suffix sequence (Table S1), which can be added by PCR using primers with 5' tails. Different types of genetic part use different prefix and suffix sequences, because they include the differing fusion sites (Table S1). Primer tail sequences for different types of genetic parts are outlined in Table S2. In each instance the prefix and suffix sequences contain the inward-facing BsaI recognition sites and corresponding storage donor fusion sites F (TGTG) and R (GACC), which are used to clone parts into Level 0 vector pStA0. Between the BsaI restriction sites are inward-facing SapI restriction sites and corresponding donor fusion sites which are not used during the storage cloning shown, but are later used for excising cloned parts from pStA0 for use in Level 1 assembly.

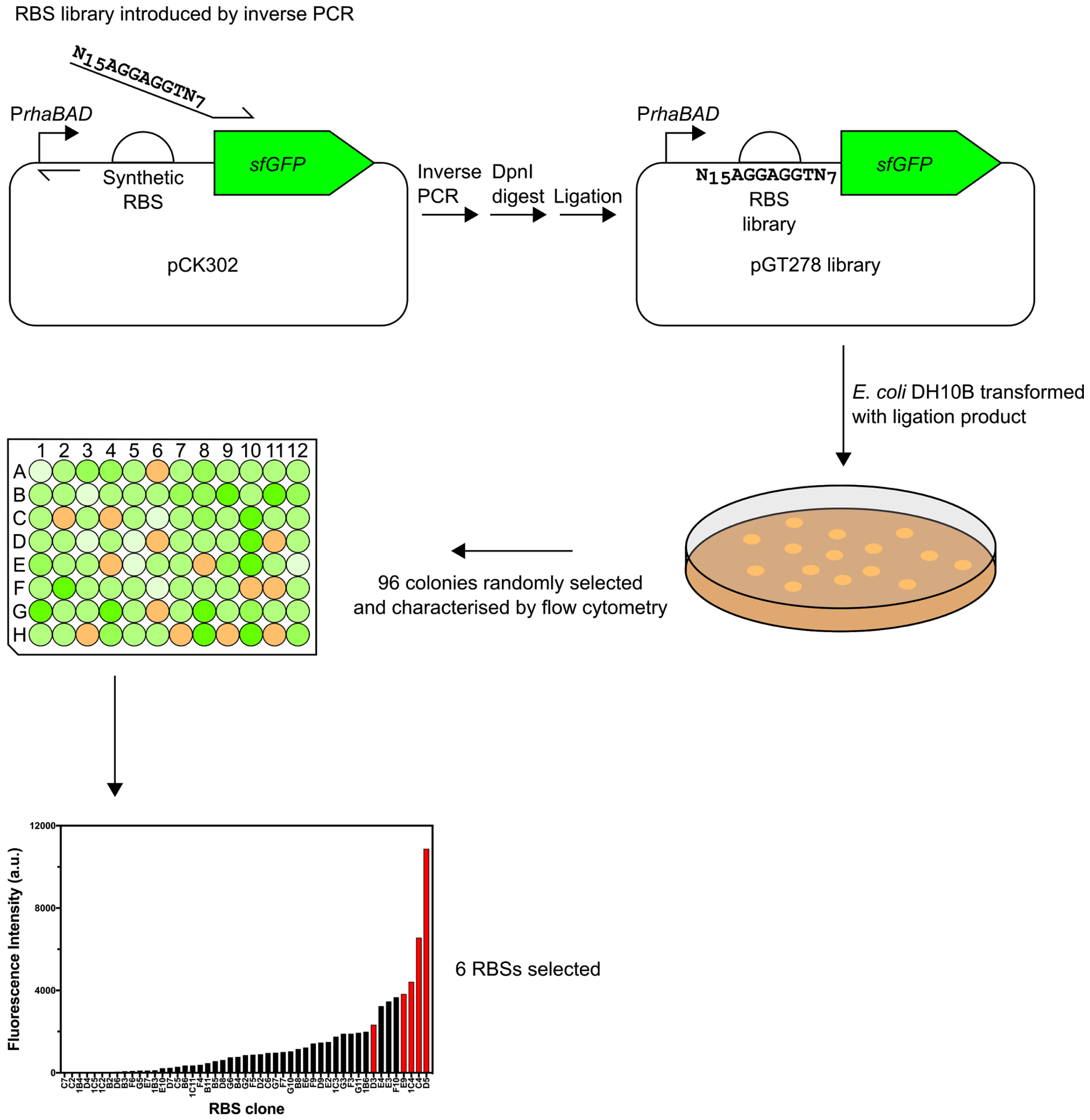


**Figure S4. Design, generation and characterisation of a synthetic RBS library.** The RBS library design conserved the *E. coli* Shine-Dalgarno (SD) consensus sequence (AGGAGGT) but randomised (N) 15 bp upstream and 7 bp downstream of the SD by incorporating the four nucleotides A, T, G and C at approximately the same 25% frequency at each N position. The RBS library was introduced to pCK302 upstream of *sfGFP* by inverse PCR using primers oligoGT448 and oligoGT463. The PCR reaction product was treated with DpnI to remove the template plasmid DNA. PCR fragments of the expected size (5.6 kbp) were excised and purified following gel electrophoresis, circularised by ligation and *E. coli* was transformed with the resultant ligation product by electroporation. 96 *E. coli* transformant colonies were randomly selected and characterised in mid-exponential phase of growth by flow cytometry (as described in Supplementary Materials and Methods - Flow cytometry analysis). The fluorescence intensity

of each clone is plotted with the background fluorescence subtracted. From this RBS library, six RBSs were chosen to give a wide and evenly-spaced distribution of expression strengths (shown in red here, and separately in Figure S5b).

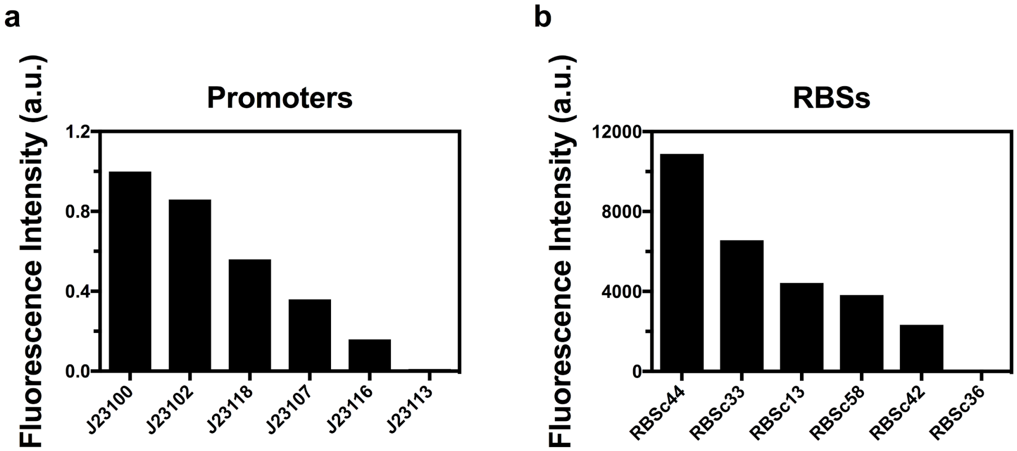


**Figure S5. Promoters and RBSs stored in pStA0 in this study. (a)** Fluorescence intensities reported by Anderson (4) for the six promoters we selected, relative to promoter J23100. **(b)** Fluorescence intensities of the six RBSs chosen from the characterised library described in Figure S4. These six RBSs give a wide and evenly-spaced distribution of expression strengths, and were stored in Level 0 vector pStA0. Promoters, in descending order of strength: P1 = J23100, P2 = J23102, P3 = J23118, P4 = J23107, P5 = J23116, P6 = J23113. RBSs, in descending order of strength: R1 = RBSc44, R2 = RBSc33, R3 = RBSc13, R4 = RBSc58, R5 = RBSc42, R6 = RBSc36.

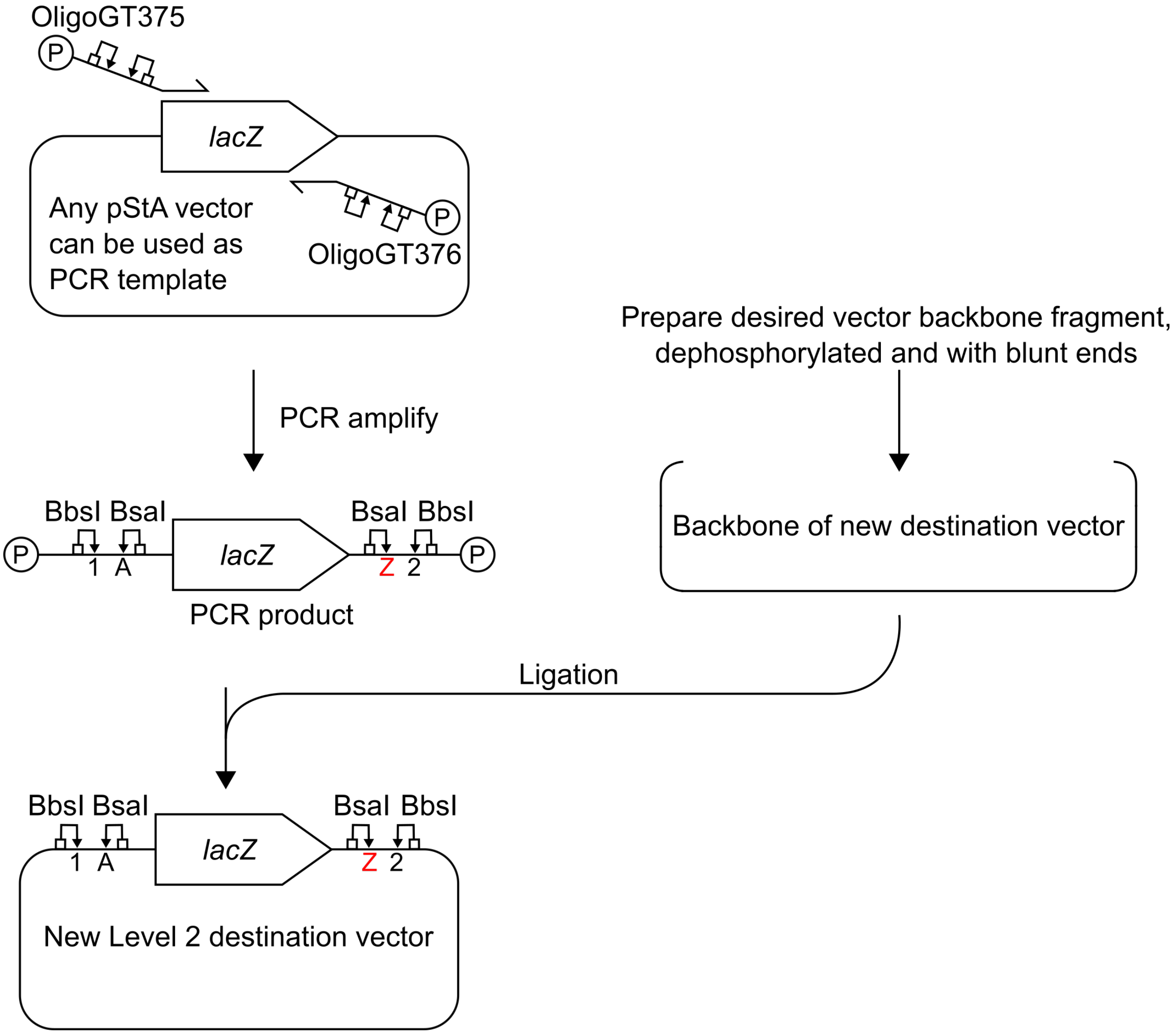


**Figure S6. Strategy for construction of an alternative Level 2 destination vector.** Prepare a linear, blunt-ended, dephosphorylated backbone fragment for the desired destination vector, for example by PCR-amplification. PCR-amplify the *lacZα* gene with primers oligoGT375 and oligoGT376 using any of the vectors in Table 1 as template. The primer tails contain outward-facing BsaI recognition sites, with corresponding A and Z acceptor fusion sites, and inward-facing BbsI recognition sites, with corresponding 1 and 2 donor fusion sites. This arrangement is the same as the assembly cassette of pStA212. Purify the PCR product and ligate it with the backbone vector fragment. Ligation of blunt-ended DNA fragments is not orientation-specific. Transform a suitable cloning strain of *E. coli* with the ligation product and use blue/white screening to identify blue colonies containing the desired plasmid with the assembly cassette. Verify the plasmid by DNA sequencing. BsaI sites and/or BbsI sites in the vector backbone should be removed.

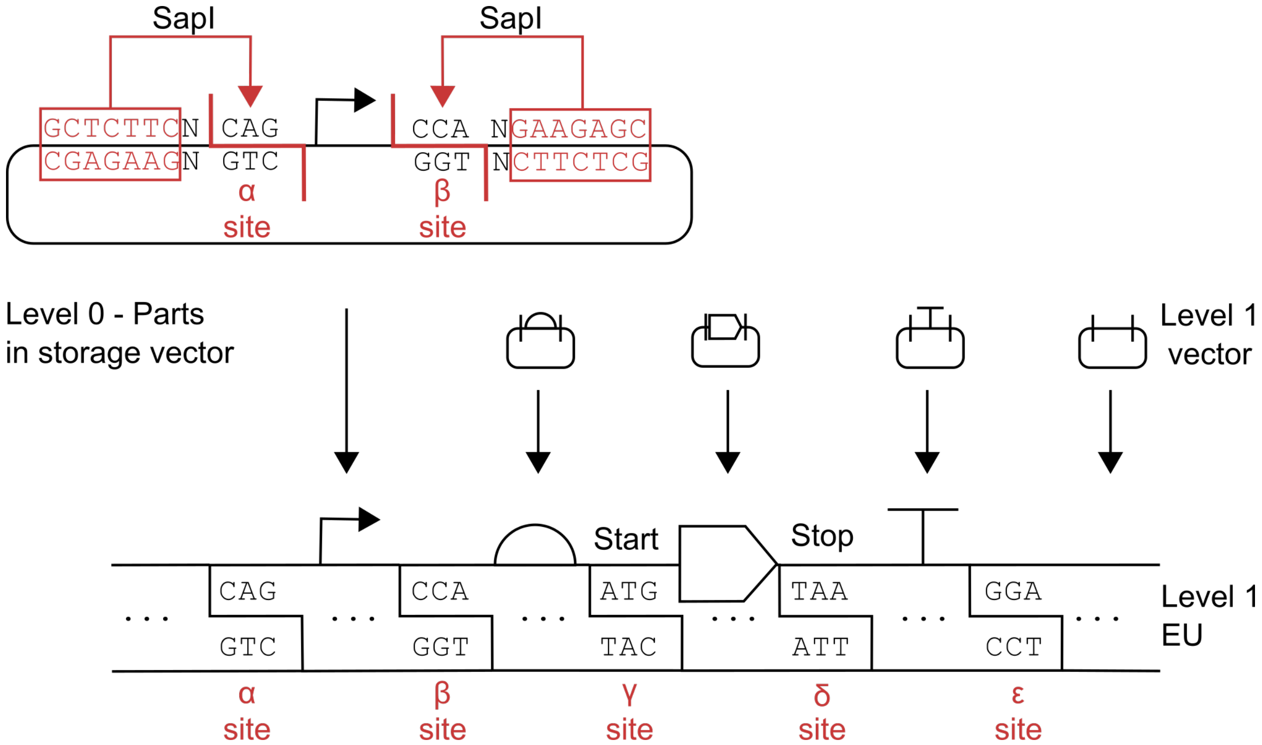


**Figure S7. Level 1 assembly of an expression unit showing detail of Level 0 promoter part.** Genetic parts stored in pStA0 are flanked by inward-facing SapI recognition sites (red boxes) with corresponding donor fusion sites (staggered red lines) for Level 1 assembly of expression units. Promoters use the α site (CAG) and β site (CCA).

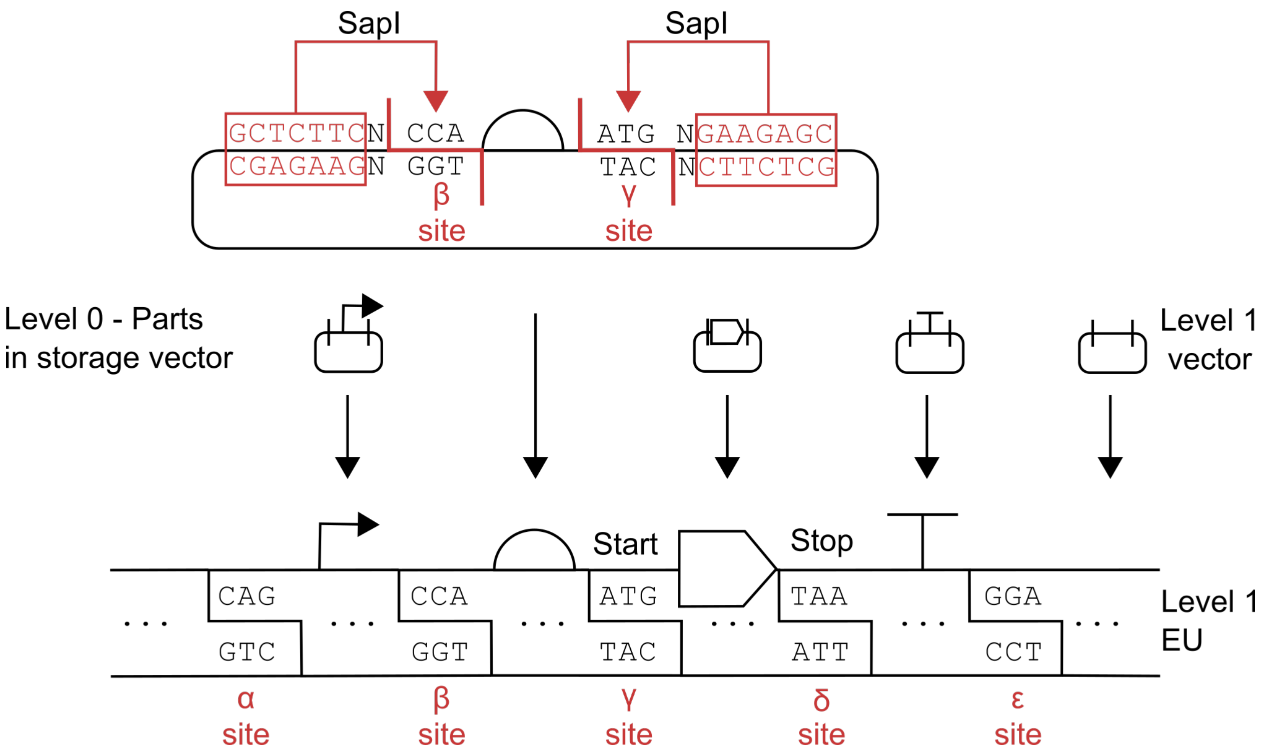


**Figure S8. Level 1 assembly of an expression unit showing detail of Level 0 RBS part.** Genetic parts stored in pStA0 are flanked by inward-facing SapI recognition sites (red boxes) with corresponding donor fusion sites (staggered red lines) for Level 1 assembly of expression units. RBSs use the β site (CCA) and γ site (ATG, start codon).

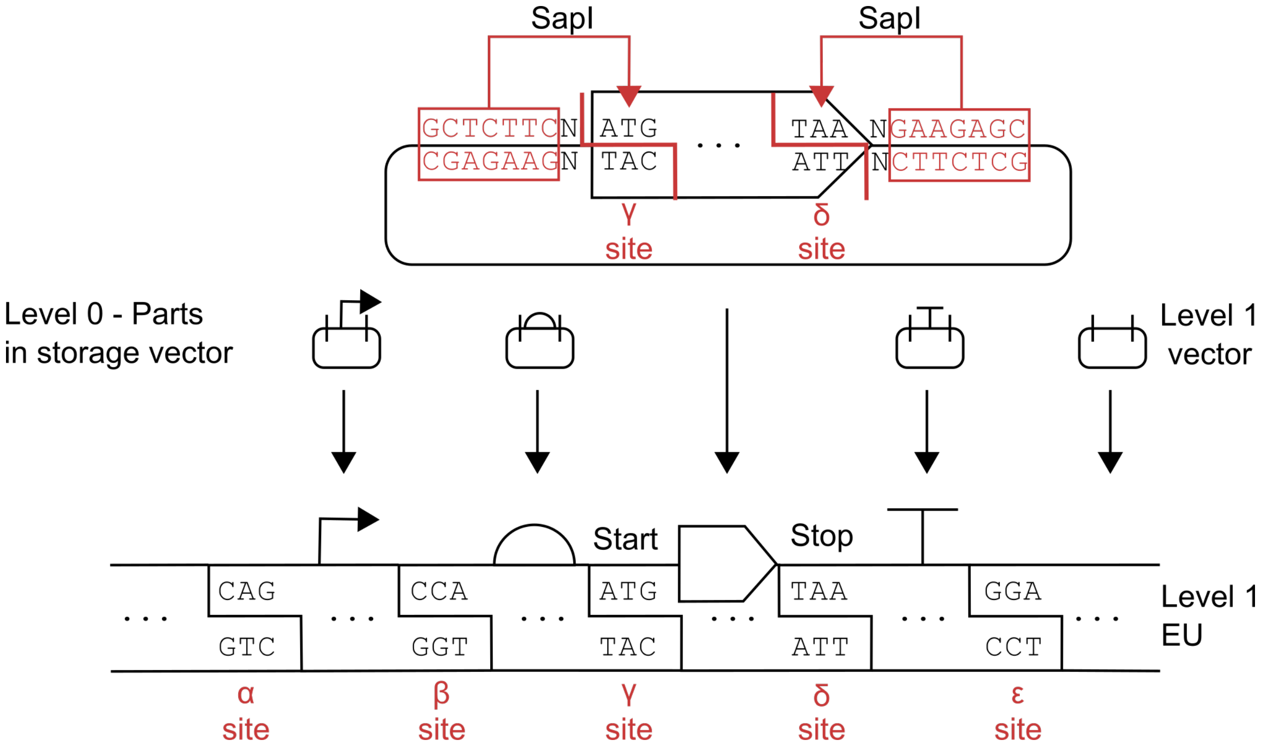


**Figure S9. Level 1 assembly of an expression unit showing detail of Level 0 CDS part.** Genetic parts stored in pStA0 are flanked by inward-facing SapI recognition sites (red boxes) with corresponding donor fusion sites (staggered red lines) for Level 1 assembly of expression units. CDSs use the γ site (ATG, start codon) and δ site (TAA, stop codon).

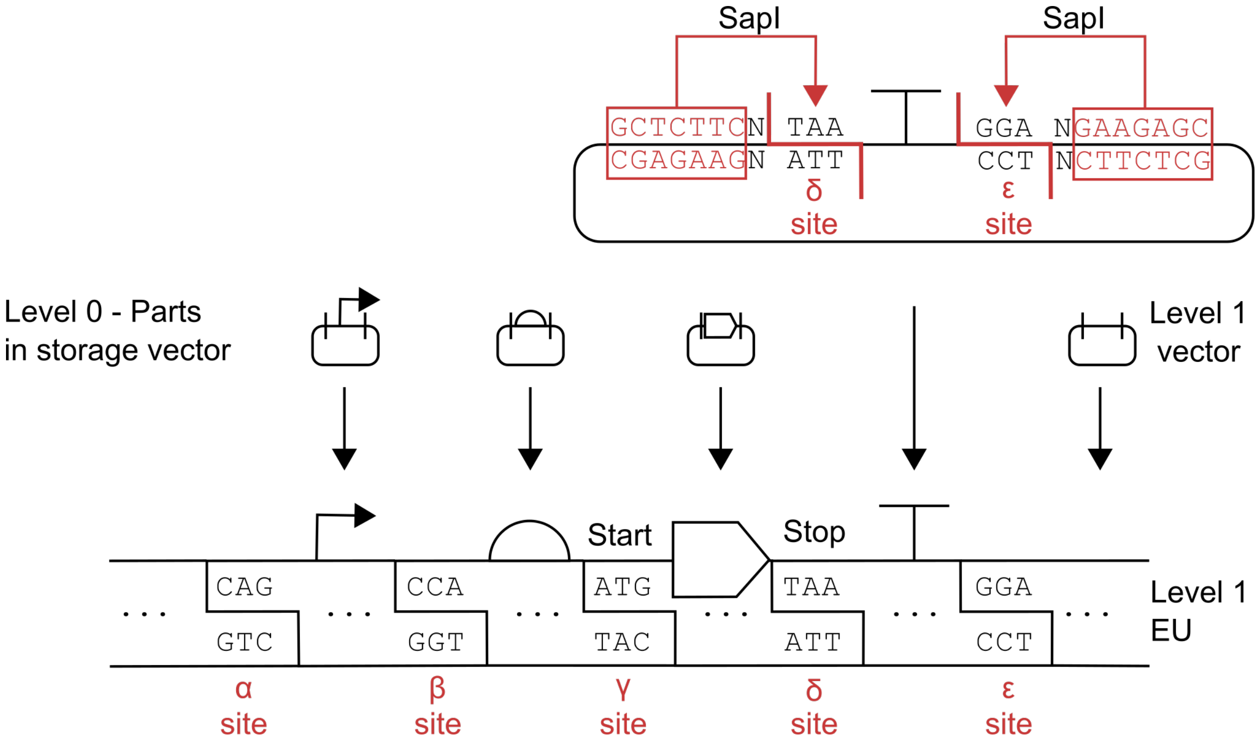


**Figure S10. Level 1 assembly of an expression unit showing detail of Level 0 terminator part.** Genetic parts stored in pStA0 are flanked by inward-facing SapI recognition sites (red boxes) with corresponding donor fusion sites (staggered red lines) for Level 1 assembly of expression units. Terminators use the δ site (TAA, stop codon) and ε site (GGA).

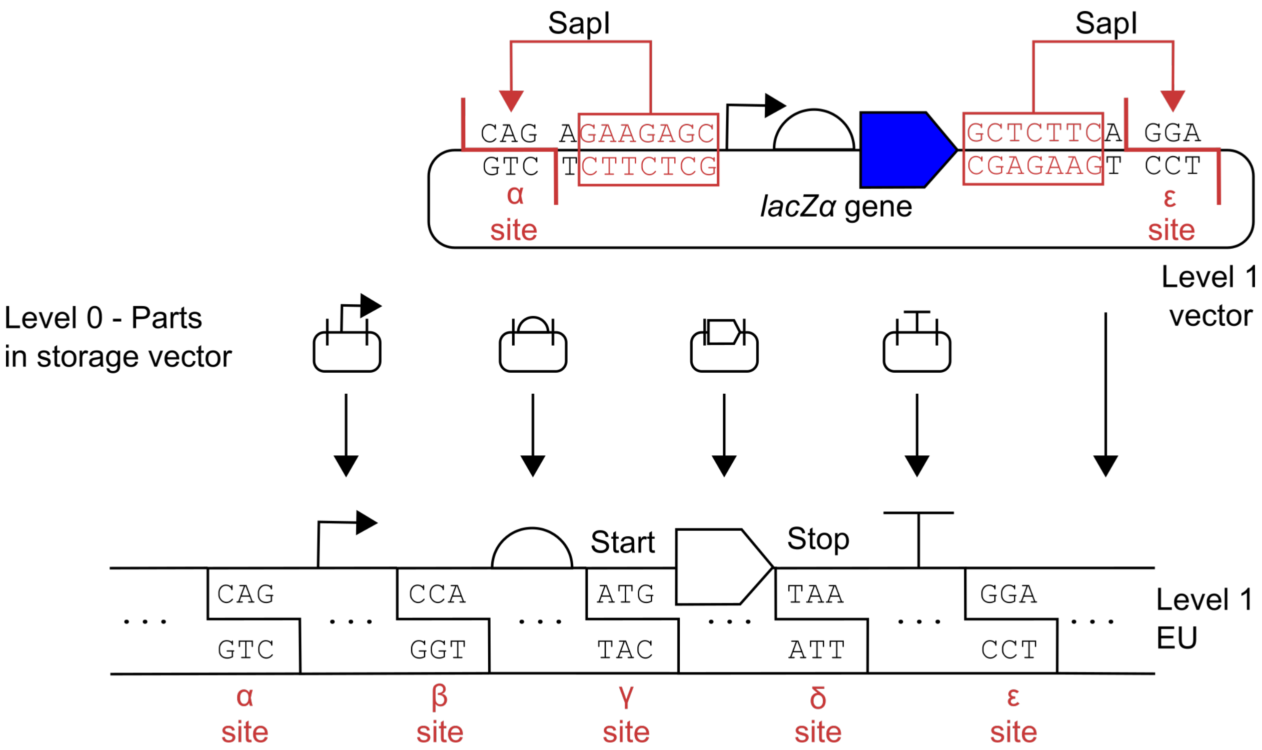


**Figure S11. Level 1 assembly of an expression unit showing detail of Level 1 vector.** Level 1 vectors contains two outward-facing SapI recognition sites (red boxes) with corresponding α (CAG) and ε (GGA) acceptor fusion sites (staggered red lines) for Level 1 assembly of expression units. Between the SapI sites in a Level 1 empty vector is a *lacZα* gene which can be used for blue/white screening.

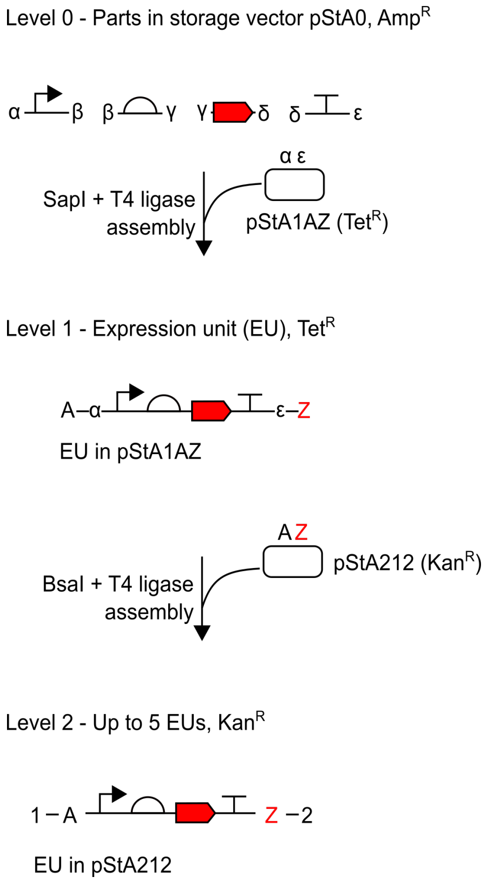


**Figure S12. General strategy for assembling one expression unit using Start-Stop Assembly.** One expression unit is assembled in Level 1 vector pStA1AZ from Level 0 parts by Level 1 assembly using the fusion sites α, β, γ, δ and ε. For cases in which only a single expression unit is required, either a Level 1 vector can be used directly for expression, or the expression unit can be transferred to a Level 2 destination vector via a Level 2 assembly reaction.

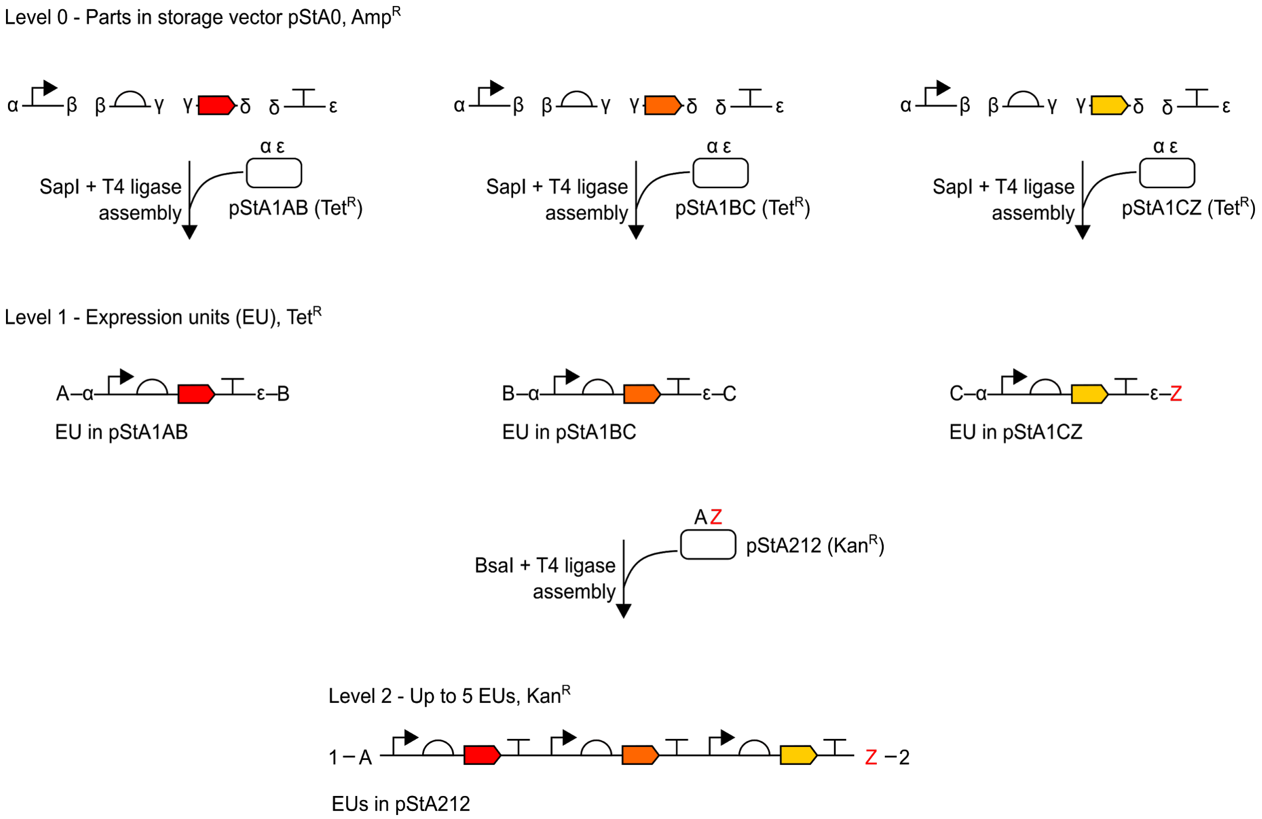


**Figure S13. General strategy for assembling three expression units in a single construct using Start-Stop Assembly.** Three expression units are independently assembled in the Level 1 vectors pStA1AB, pStA1BC and pStA1CZ from Level 0 parts by Level 1 assembly using the fusion sites α, β, γ, δ and ε. The three expression units can be assembled as a single construct in a Level 2 vector (in this case pStA212) by Level 2 assembly using the fusion sites A, B, C and Z.

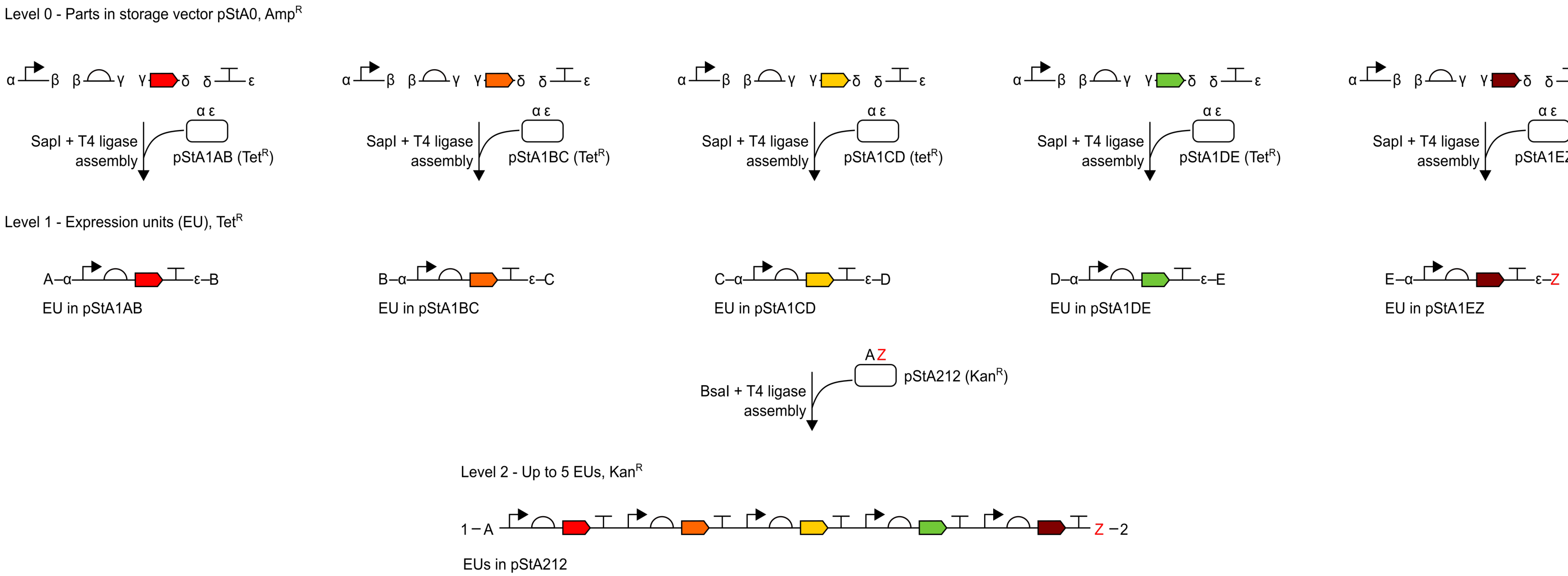


**Figure S14. General strategy for assembling five expression units in a single construct using Start-Stop Assembly.** Five expression units are independently assembled in the Level 1 vectors, pStA1AB, pStA1BC, pStA1CD, pStA1DE and pStA1EZ from Level 0 parts by Level 1 assembly using the fusion sites α, β, γ, δ and ε. The five expression units can be assembled as a single construct in a level 2 vector (in this case pStA212) by Level 2 assembly using the fusion sites A, B, C, D, E and Z.

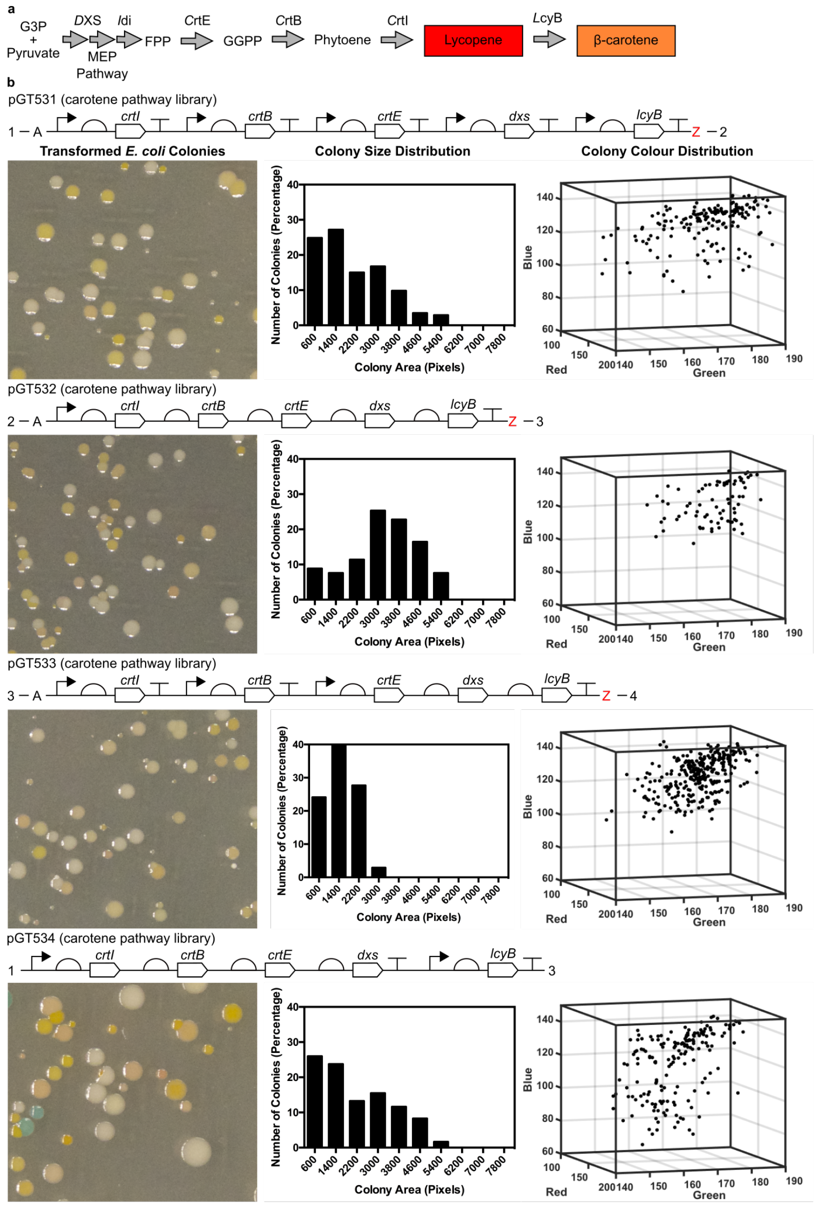


**Figure S15. Analysis of the four β-carotene pathway libraries. (a)** β-carotene pathway showing the endogenous enzymes of the *E. coli* MEP pathway and heterologous enzymes. Coloured products are shown in boxes, enzymes are shown above arrows. Abbreviations: G3P (glyceraldehyde 3-phosphate), FPP (Farnesyl pyrophosphate), GGPP (Geranylgeranyl pyrophosphate). **(b)** Designs of β-carotene pathway libraries (pGT531-534) and phenotypic variation among *E. coli* clones from those libraries. Variation was compared between the four β-carotene combinatorial pathway libraries (pGT531-534) and the control plasmids, pStA314 and pGT536 (data for the controls is shown in Figure 5c). Phenotypic variation is shown using representative pictures of the transformation plates, histograms of colony size (measured as cell area, *x*-axis represents the upper limit of each histogram bin) and the distributions of colony colours (measured using colony red, green and blue values extracted from colony images).

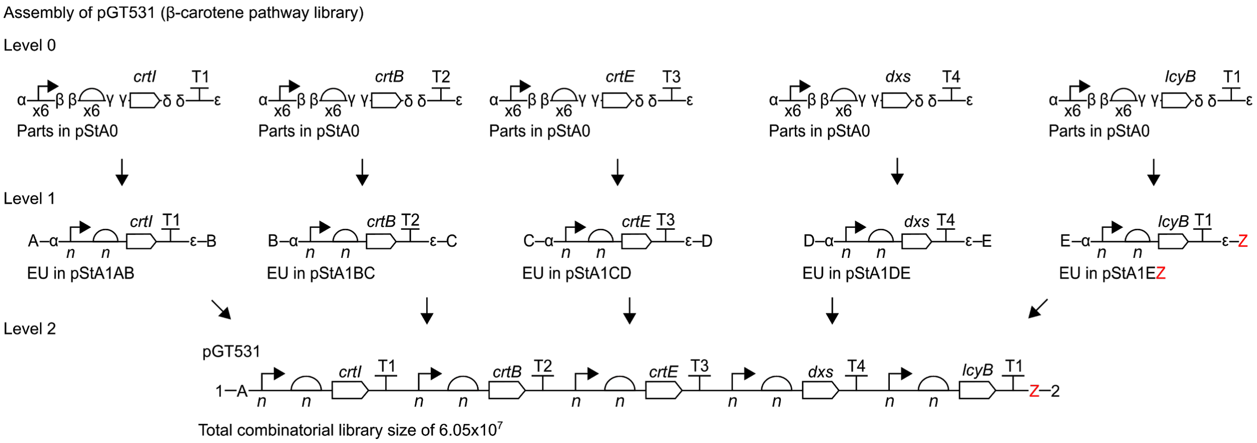


**Figure S16. Combinatorial assembly of monocistronic β-carotene pathway library pGT531.** A library of constructs each encoding a β-carotene pathway was assembled hierarchically and combinatorially as shown in the figure and as described in the main text. The destination vector was pStA212. Equimolar mixtures of six promoter parts or six RBS parts (Figure S5) labelled as 'x6' were used in Level 1 assemblies. The uncertain representation of each promoter and RBS at each position following assembly is represented by '*n*'. The maximum library size was $6^{10}$ = 6.05x$10^7$. T1 = terminator L3S2P55, T2 = terminator L3S2P21, T3 = terminator ECK120033737, T4 = terminator ECK120019600. Assembled insert size = 7,702 bp. Total size of plasmid including assembled insert = 10,255 bp.

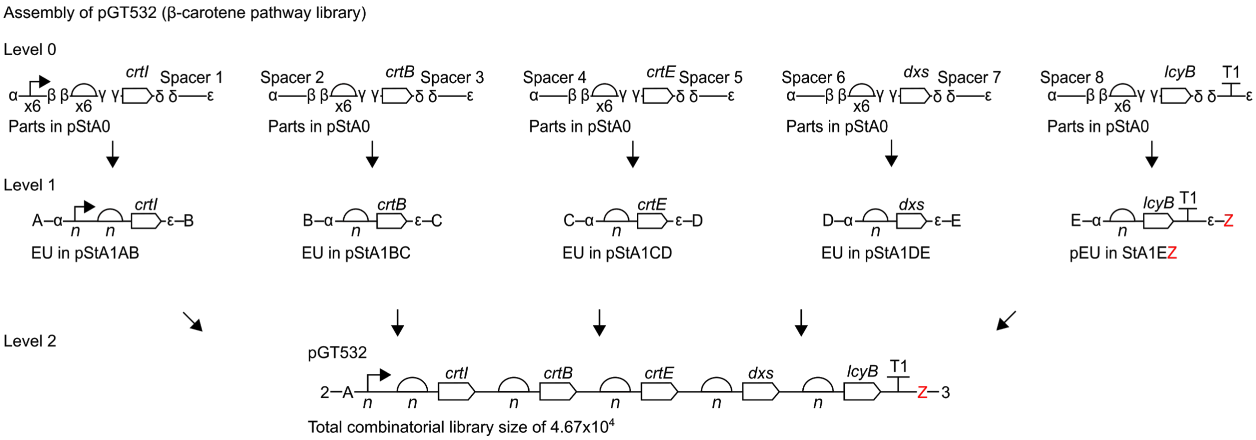


**Figure S17. Combinatorial assembly of operon-based β-carotene pathway library pGT532.** A library of constructs each encoding a β-carotene pathway was assembled hierarchically and combinatorially as shown in the figure and as described in the main text. The destination vector was pStA223. Equimolar mixtures of six promoter parts or six RBS parts (Figure S5) labelled as 'x6' were used in Level 1 assemblies where shown. Spacer sequences were used in Level 1 assemblies (as linkers, Table S5) in place of promoters and/or terminators where shown. The uncertain representation of each promoter and RBS at each position following assembly is represented by '*n*'. The maximum library size was $6^6 = 4.6x10^4$. T1 = terminator L3S2P55. Assembled insert size = 7,393 bp. Total size of plasmid including assembled insert = 9,946 bp.

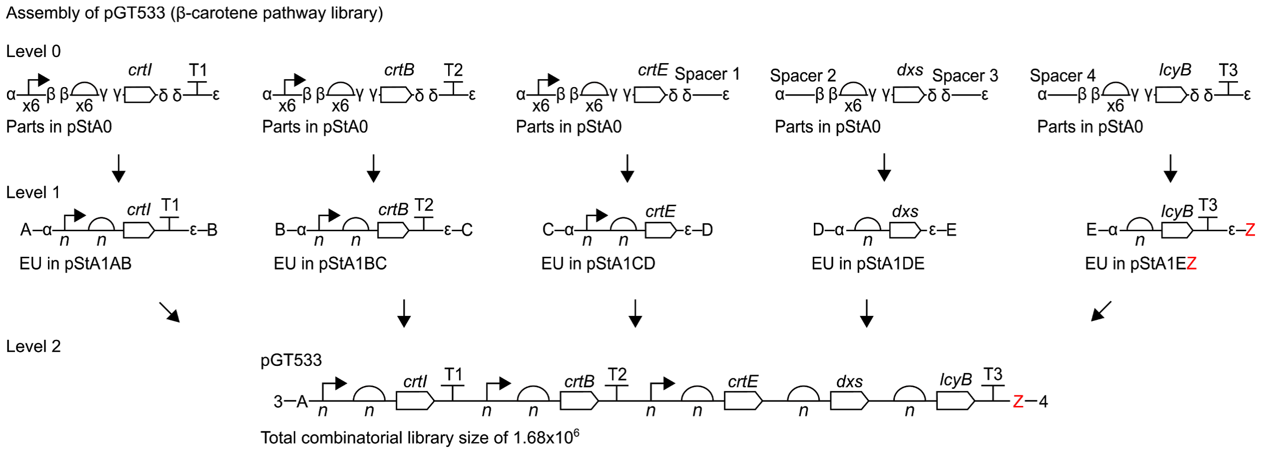


**Figure S18. Combinatorial assembly of hybrid β-carotene pathway library pGT533**. A library of constructs each encoding a β-carotene pathway was assembled hierarchically and combinatorially as shown in the figure and as described in the main text. The destination vector was pStA234. Equimolar mixtures of six promoter parts or six RBS parts (Figure S5) labelled as 'x6' were used in Level 1 assemblies where shown. Spacer sequences were used in Level 1 assemblies (as linkers, Table S5) in place of promoters and/or terminators where shown. The uncertain representation of each promoter and RBS at each position following assembly is represented by '*n*'. The maximum library size was $6^8 = 1.68 \times 10^6$. T1 = terminator L3S2P55, T2 = terminator L3S2P21, T3 = terminator ECK120033737. Assembled insert size = 7,533 bp. Total size of plasmid including assembled insert = 10,086 bp.

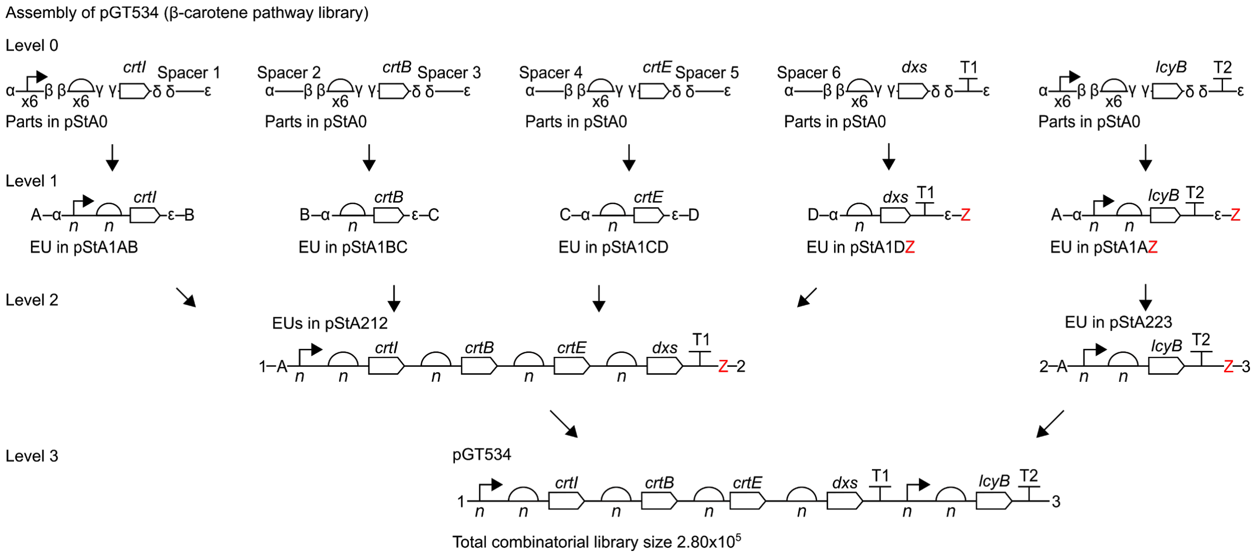


**Figure S19. Combinatorial assembly of hybrid β-carotene pathway library pGT534**. A library of constructs each encoding a β-carotene pathway was assembled hierarchically and combinatorially as shown in the figure and as described in the main text. The destination vector was pStA313. Equimolar mixtures of six promoter parts or six RBS parts (Figure S5) labelled as 'x6' were used in Level 1 assemblies where shown. Spacer sequences were used in Level 1 assemblies (as linkers, Table S5) in place of promoters and/or terminators where shown. The uncertain representation of each promoter and RBS at each position following assembly is represented by '*n*'. The maximum library size was $6^7 = 2.80 \times 10^5$. T1 = terminator L3S2P55, T2 = terminator L3S2P21. Assembled insert size = 7,481 bp. Total size of plasmid including assembled insert = 10,188 bp.

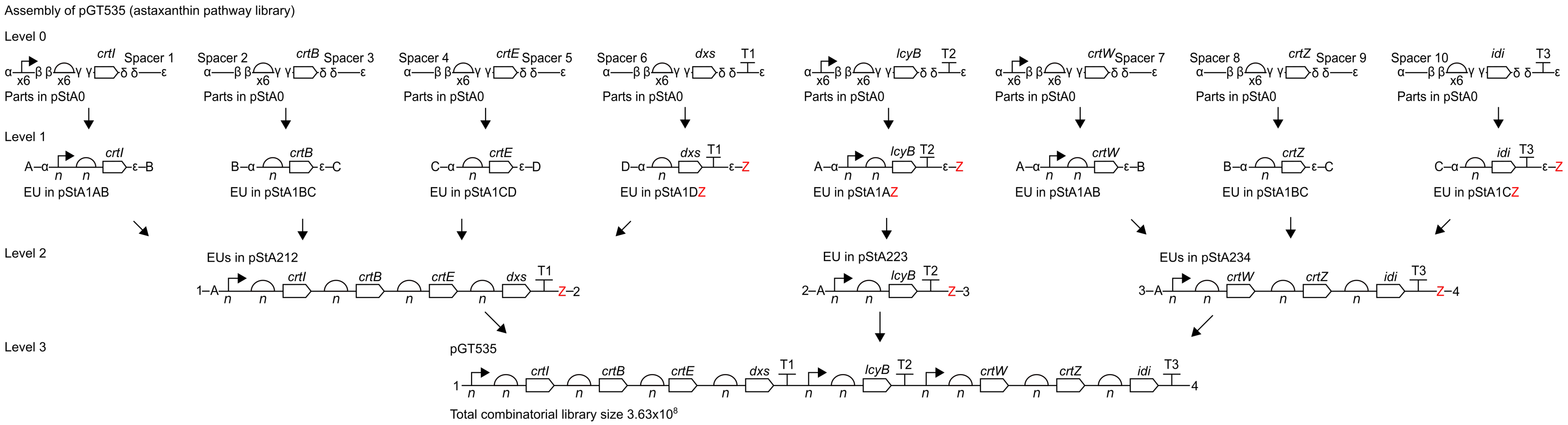


**Figure S20. Assembly of pGT535; astaxanthin pathway library in pStA314.** Illustration of the combinatorial assembly of the eight coding sequence astaxanthin pathway in pStA314. A library of six promoters (J23100, J23102, J23118, J23107, J23116, J23113; shown as ‘x6’) and a library of six RBSs (RBSc44, RBSc33, RBSc13, RBSc58, RBSc42, RBSc36; shown as ‘x6') were used for the combinatorially assembly. To assemble operons predetermined spacers were substituted for promoters and terminators as needed. The coding sequence *crtI* was combinatorially assembled with the libraries of promoters and RBSs in pStA1AB and spacer 1 (δ-ε). The coding sequence *crtB* was combinatorially assembled with the library of RBSs in pStA1BC and spacer 2 (α-β) and 3 (δ-ε) to replace the promoter and terminator, respectively. The coding sequence *crtE* was combinatorially assembled with the library of RBSs in pStA1CD with spacers 4 (α-β) and 5 (δ-ε) to replace the promoter and terminator, respectively. The coding sequence *dxs* was combinatorially assembled with the RBS library in pStA1DZ with spacers 6 (α-β) and the terminator L3S2P55 (T1). These four expression units were subsequently assembled as a four coding sequence operon at Level 2 in pStA212. The coding sequence *lcyB* was combinatorially assembled as a monoscitronic at Level 1 with the promoter and RBS libraries in pStA1AZ and terminator L3S2P21 (T2), this expression unit was then assembled in pStA223. The coding sequence *crtW* was combinatorially assembled with the library of promoters and RBSs in pStA1AB with spacer 7 (δ-ε), *crtZ* was assembled with the RBS library and spacers 8 (α-β) and 9 (δ-ε) to replace the promoter and terminator, respectively. The final coding sequence *idi* was assembled with spacer 10 (α-β), the RBS library and terminator ECK120033737 (T3) in pStA1CZ, these three expression units (crtW, crtZ and idi) were then assembled as a three coding sequence operon in pStA234. All eight expression units were finally combinatorially assembled at Level 3 in pStA314. This astaxanthin pathway variant had a total combinatorial library size of 362,797,056, a result of multiplying the number of parts in each combinatorial position (represented as ‘*n*’). More specifically, upstream of *crtI*, *lcyB* and *crtW* was a library of 6 promoters and 6 RBSs and for *crtB*, *crtE*, *dxs*, *crtZ* and *idi* a library of 6 RBSs therefore $6^{11} = 3.6\text{x}10^{8}$. Assembled insert size = 9,984 bp. Total size of plasmid including assembled insert = 12,691 bp.

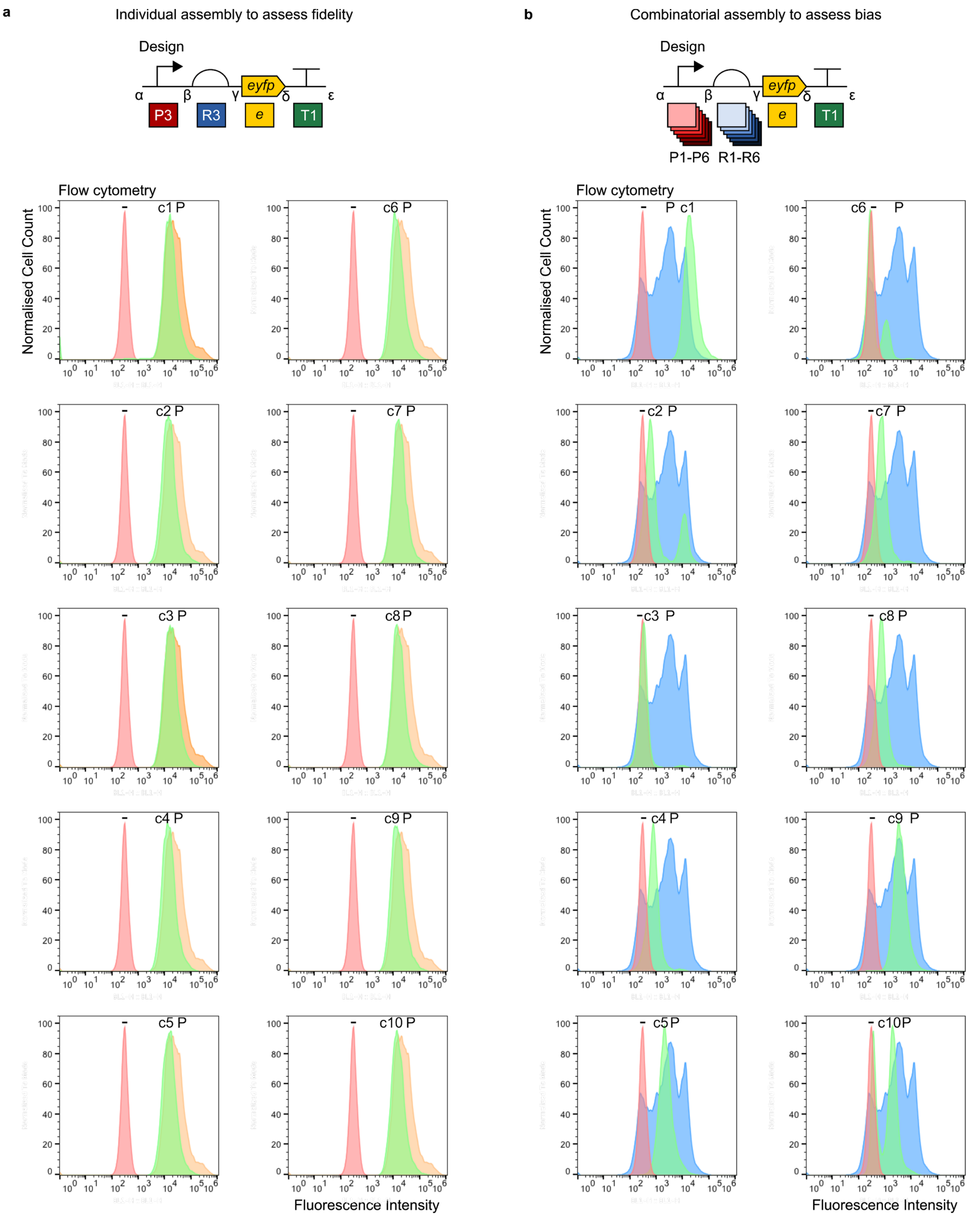


**Figure S21. Flow cytometry data for individual clones used in assessment of fidelity and bias of multi-part Level 1 assembly.** The experiments are described in the main text and Figure 2. Flow cytometry histograms show fluorescence intensity of 10,000 events (cells)

normalised to the maximum (in order to visualise distribution rather than absolute values) for wild-type *E. coli* DH10B as a negative reference (-), each of the ten clones (c1-c10) and a pool of several hundred transformants (P). **(a)** Assessment of assembly fidelity using assembly of an individual P3-R3-*e*-T1 expression unit. The ten clones showed similar fluorescence intensity to one another. **(b)** Assessment of assembly bias by combinatorial assembly of EYFP expression units using six promoters P1-P6, six RBSs R1-R6, *eyfp* and terminator T1. The ten clones showed widely differing fluorescence intensity values.

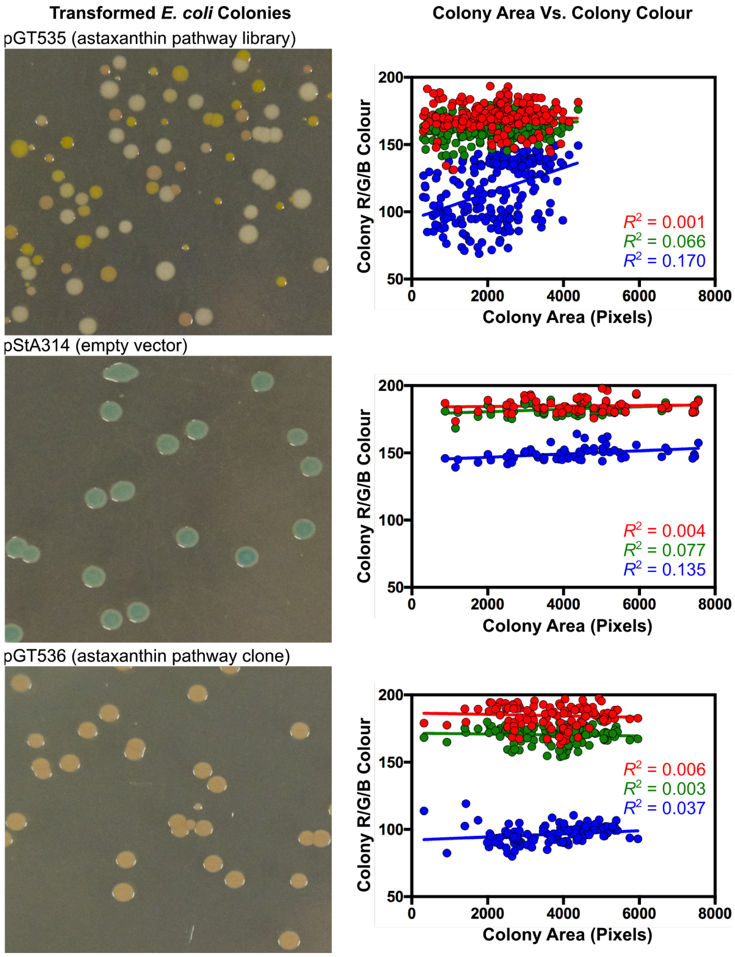


**Figure S22. Relationship between colony area and colony colour in the astaxanthin pathway library.** The experiment is described in the main text and Figure 5. Phenotypic variation among *E. coli* clones of the astaxanthin pathway library (pGT535) was compared to controls pStA314 (empty vector) and an isolated clone pGT536 from the astaxanthin pathway library pGT535. The representative images of colonies shown are the same as those in Figure 5c. Colony area and RGB colour (red, green, blue) values were extracted from images. For each colony, the three colour values are plotted separately in the corresponding colour. Linear regressions for each R/G/B colour are also shown in the corresponding colour (solid lines and $R^2$ values). There is little or no association between colony area and colony colour, reflected by low $R^2$ values.

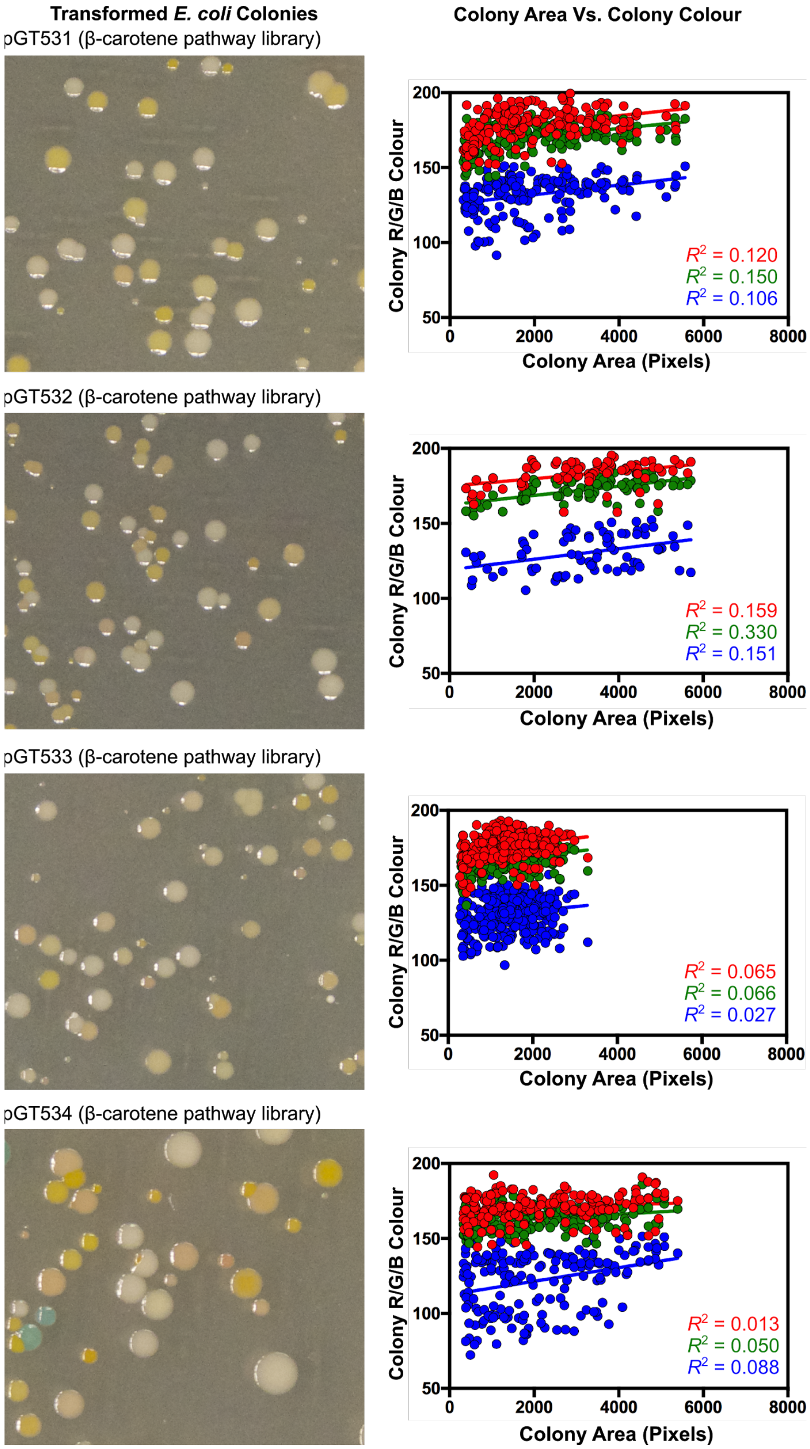


**Figure S23. Relationship between colony area and colony colour in the β-carotene pathway libraries.** The experiment is described in the main text and Figure S15. The representative images of colonies shown are the same as those in Figure 15. Colony area and RGB colour (red, green, blue) values were extracted from images. For each colony, the three colour values are plotted separately in the corresponding colour. Linear regressions for each R/G/B colour are also shown in the corresponding colour (solid lines and $R^2$ values). There is little or no association between colony area and colony colour, reflected by low $R^2$ values.

Combinatorial assembly to assess bias

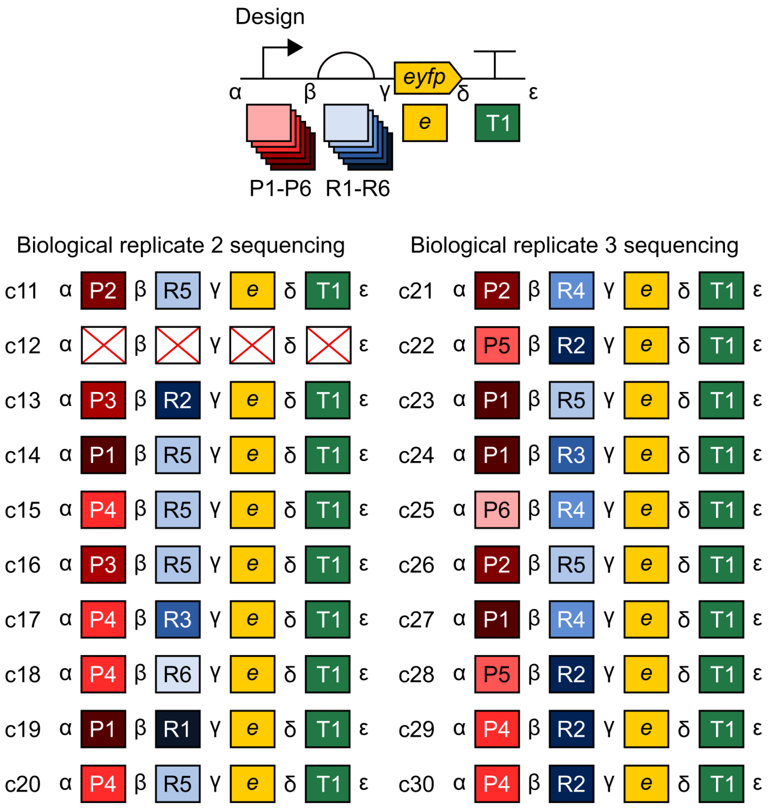


Consistent with unbiased assembly

**Figure S24. Sequencing results of two additional biological replicates of the experiment to assess of bias of multi-part Level 1 assembly.** Constructs were assembled from Level 0 parts (in storage plasmids) into Level 1 vector pStA1AZ, as described in Figure 2b. Promoters, in descending order of strength: P1 = J23100, P2 = J23102, P3 = J23118, P4 = J23107, P5 = J23116, P6 = J23113. RBSs, in descending order of strength: R1 = RBSc44, R2 = RBSc33, R3 = RBSc13, R4 = RBSc58, R5 = RBSc42, R6 = RBSc36. CDS: *e* = *eyfp*. Terminator: T1 = L3S2P55. Assessment of assembly bias by combinatorial assembly of EYFP expression units using six promoters P1-P6, six RBSs R1-R6, *eyfp* and terminator T1. Ten colonies from each assembly were randomly selected for analysis. The red crosses indicates a misassembly between α and ε in place of the expression unit.

## SUPPLEMENTARY NOTES

### Note S1: Start-Stop Assembly Quick-Start Guide

See separate file.

### Note S2. Cloning parts into Level 0 vector pStA0

We suggest three approaches for cloning parts into Level 0 vector pStA0:

1. **Inverse PCR adding part sequence using primer tails**. Use pStA0 as the PCR template and primers which anneal outside the BsaI sites of pStA0 (removing the BsaI sites and *lacZα* from resultant construct). See Table S8 for primer annealing regions and SapI sites and corresponding fusion sites to be included in primer tails. The part being cloned into pStA0 is added using primer tail(s) outside the SapI sites and fusion sites. The part can be added to one primer or split between both to avoid excessively long primers. Use a DNA polymerase which yields blunt DNA ends, suitable for ligation. At least one end of the PCR product must include a 5' phosphate group to allow ligation, which is most conveniently achieved by obtaining a primer with a 5' phosphate group from the manufacturer. This approach is particularly useful for small parts and when no template for the part is available.

2. **PCR amplification followed by one-pot assembly with pStA0**. See Figure S3. PCR-amplify the part of interest using primers with tails shown in Table S2. The purified PCR product can be used directly in a Level 0 one-pot assembly reaction with pStA0 as described in Materials and Methods.

3. **DNA synthesis followed by one-pot assembly with pStA0**. Design the part with prefix and suffix sequences shown in Table S1. The synthesised DNA fragment can be used directly in a Level 0 one-pot assembly reaction with pStA0 as described in Materials and Methods.

### Note S3: Start-Stop Assembly Lab Protocol

See separate file.

## SUPPLEMENTARY MATERIALS AND METHODS

### Plasmid construction

Plasmids, oligonucleotides and synthetic DNA used in this study are shown in Tables 1, S2, S3 and S5. All oligonucleotides and gBlock linear DNA fragments were synthesised by Integrated DNA Technologies Inc. All Start-Stop Assembly vector sequences have been deposited in GenBank (accession numbers shown in Table 1 and Table S3).

### Construction of Level 0 vector pStA0

Level 0 vector pStA0 was derived from pUC19. To silence a BsaI site in the ampicillin-resistance cassette the pUC19 backbone was amplified in two fragments, the first using primers oligoGT249 and oligoGT252, and the second using oligoGT250 and oligoGT251. OligoGT251 and oligoGT252 include overlapping regions with a single nucleotide mutation to silence the BsaI site. The two PCR products served as a template for overlap extension PCR (5) using oligoGT249 and oligoGT250 to generate a blunt-ended vector backbone fragment lacking the BsaI site. This vector backbone PCR product was ligated with 5'-phosphorylated gblock dnaGT6 (obtained from the supplier with 5' phosphate groups already in place) to generate pGT421. The pGT421 backbone was PCR-amplified using oligoGT253 and oligoGT254. The *lacZα* fragment was amplified from pUC19 using oligoGT255 and oligoGT256 and phosphorylated using T4 Polynucleotide Kinase. The pGT421 backbone and phosphorylated *lacZα* fragment were ligated together to generate pStA0.

### Construction of Level 1 vectors

The nine Level 1 vectors (Table 1) were derived from pACYC184. To silence a BbsI site in the tetracycline-resistance cassette the pACYC184 backbone was amplified in two fragments, the first using primers oligoGT257 and oligoGT259, and the second using oligoGT258 and oligoGT260. OligoGT259 and oligoGT260 include overlapping regions with a single nucleotide mutation to silence the BbsI site. The two PCR products served as a template for overlap extension PCR using oligoGT257 and oligoGT258 to generate a blunt-ended vector backbone fragment lacking the BbsI site. This vector backbone PCR product was ligated with a *lacZα* fragment amplified from pUC19 using oligoGT261 and oligoGT262 to generate pGT422.

Plasmids pStA1AZ, pStA1BC, pStA1CD and pStA1DE were generated using overlap extension PCR and pGT422 as the template. The pStA1AZ *lacZα* fragment was amplified using oligoGT356 and oligoGT357 and the backbone was amplified using oligoGT265 and oligoGT266. The pStA1BC *lacZα* fragment was amplified using oligoGT363 and oligoGT366 and the backbone was amplified using oligoGT268 and oligoGT270. The pStA1CD *lacZα* fragment was amplified using oligoGT364 and oligoGT367 and the backbone was amplified using oligoGT268 and oligoGT273. The pStA1DE *lacZα* fragment was amplified using oligoGT365 and oligoGT368 and the backbone was amplified using oligoGT268 and oligoGT276. Each pair of overlapping PCR products was extended by overlap extension PCR to

generate a single PCR product, which was subsequently phosphorylated and ligated using the NEB site-directed mutagenesis kit to generate the corresponding complete vector.

Plasmids pStA1AB, pStA1BZ, pStA1CZ, pStA1DZ and pStA1EZ were generated using inverse PCR and pStA1AZ as the PCR template. pStA1AB was generated using oligoGT268 and oligoGT362, pStA1BZ was generated using oligoGT279 and oligoGT358, pStA1CZ was generated using oligoGT279 and oligoGT359, and pStA1DZ was generated using oligoGT279 and oligoGT360. PCR products were ligated to obtain circular vectors.

**Construction of Level 2 vectors**

The three Level 2 vectors (Table 1) were derived from pACYC177. First, the pACYC177 backbone was PCR-amplified using primers oligoGT482 and oligoGT483, and ligated with a *lacZα* fragment amplified from pStA0 using primers oligoGT261 and oligoGT262, to generate pGT410. Next, the three Level 2 vectors were each generated by overlap extension PCR using pGT410 as template. For pGT411 the *lacZα* fragment was amplified with oligoGT377 and oligoGT378 and the backbone was amplified with oligoGT289 and oligoGT265. For pGT412 the *lacZα* fragment was amplified with oligoGT379 and oligoGT381 and the backbone was amplified with oligoGT380 and oligoGT265. For pGT413 the *lacZα* fragment was amplified with oligoGT382 and oligoGT384 and the backbone was amplified with oligoGT265 and oligoGT383. Each pair of overlapping PCR products was extended by overlap extension PCR to generate a single PCR product, which was subsequently phosphorylated and ligated using the NEB site-directed mutagenesis kit to generate the corresponding complete vector.

pGT411, pGT412 and pGT413 had a BsaI site in the backbone that was silenced via inverse PCR using primers oligoGT569 and oligoGT570. The PCR products were each phosphorylated and ligated using the NEB site-directed mutagenesis kit to generate final three Level 2 plasmids pStA212, pStA223 and pStA234.

**Construction of Level 3 vectors**

The Level 3 vectors (Table 1) were derived from pACYC184. First the vector backbone was PCR-amplified from pACYC184 using oligoGT297 and oligoGT298 and ligated with a *lacZα* fragment amplified from pStA0 using oligoGT261 and oligoGT262 to generate pGT414. To construct pStA313, the vector backbone of pGT414 was PCR-amplified using primers oligoGT265 and oligoGT299, and the *lacZα* fragment was PCR-amplified using primers oligoGT385 and oligoGT386. The two overlapping PCR products were extended by overlap extension PCR to generate a single PCR product, which was subsequently phosphorylated and ligated using the NEB site-directed mutagenesis kit to generate the vector pStA313.

Vector pStA314 was generated by inverse PCR using primers oligoGT265 and oligoGT387 with pStA313 as the template. The PCR product was phosphorylated and ligated using the NEB site-directed mutagenesis kit to generate the vector pStA314.

**Parts cloned into pStA0**

To construct pStA0::*eyfp* the *eyfp* coding sequence was PCR-amplified from the reporter plasmid pATM2 (6) using primers oligoGT614 and oligoGT615. The gel-purified PCR product was directly used in a Level 0 Start-Stop Assembly reaction with pStA0. The Level 0 reaction product was then used to transform *E. coli* and the plasmids of isolated transformant colonies were purified and sequence verified. To generate the eight plasmids pStA0::*crtI*, pStA0::*crtB*, pStA0::*crtE*, pStA0::*dxs*, pStA0::*crtW*, pStA0::*crtZ*, pStA0::*lcyB* and pStA0::*idi* codon-optimised CDSs (Table S6) were synthesised as linear gBlock DNA fragments (Integrated DNA Technologies) and cloned into pStA0. Linear gBlock DNA fragments were used directly in Level 0 Start-Stop Assembly reactions with pStA0. The Level 0 reaction product was then used to transform *E. coli* and the plasmids of transformant colonies were purified and sequence verified.

The promoters, RBSs and terminators sequences were cloned into pStA0 by inverse PCR using pStA0 as the template and the primers shown in Table S7 (primer tails correspond to sequence of genetic part). The PCR products were phosphorylated and ligated using the NEB site-directed mutagenesis kit and *E. coli* was transformed with the ligation product.

**Additional plasmids used in study**

pGT274, pGT277, pGT550 and pGT552 were generated by inverse PCR using pCK302 (1) as a template. pGT274 was constructed by PCR-amplification of pCK302 using degenerate primers oligoGT468 and oligoGT472. The PCR product was phosphorylated and ligated using the NEB site-directed mutagenesis kit and *E. coli* was transformed with the ligation product. pGT274 was isolated by randomly picking a transformant colony from the pool of clones. pGT550 was PCR amplified using oligoGT660 and oligoGT657. pGT277 was generated using oligoGT471 and oligoGT472. pGT552 was amplified using primers oligoGT660 and oligoGT659. Each of these three PCR products was phosphorylated and ligated using the NEB site-directed mutagenesis kit and *E. coli* was transformed with the ligation product. Transformant colonies were picked and sequence verified from each transformation.

**Annealing of oligonucleotide linkers**

Double-stranded linkers containing spacers were generated by annealing complementary pairs of oligonucleotides (Table S5). Pairs of oligonucleotides were mixed together to a final concentration of 10 µM. Oligonucleotide mixtures were heated to 95 °C for 5 mins, then allowed to cool slowly to room temperature. These annealed linkers were used directly as parts in Level 1 assembly reactions at a concentration of 40 fmol.

**Analysis of DNA scars and characterisation of RBS library by flow cytometry analysis**

To characterise DNA assembly scars *E. coli* DH10B cells were transformed with the appropriate individual plasmids. To characterise the RBS Library *E. coli* DH10B cells were transformed with 5 µl of RBS Library ligation product. Individual transformant colonies were used to inoculate 200

µl LB supplemented with ampicillin, which was incubated overnight (16 h) in plates of 96 U-shaped 1.2 ml wells covered with sterile breathable sealing film (Breathe Easy) at 37 °C with shaking at 700 r.p.m on a Multitron shaker (Infors-HT). For flow cytometer assays, overnight cultures were subcultured 1:1000 into 200 µl fresh LB medium that contained ampicillin and the *rhaBAD* promoter inducer L-rhamnose (0.6 mg ml-$^{1}$), in deep-well plates and grown for 6 h at 37 °C with shaking at 700 r.p.m. Cultures were diluted 1:50 in filtered PBS and immediately subjected to flow cytometer analysis. GFP fluorescence was measured using an Attune NxT flow cytometer (Invitrogen) as described in Materials and Methods.

## SUPPLEMENTARY TABLES

**Table S1. Prefix and suffix sequences**. Prefix and suffix sequences should be added to genetic parts to allow cloning into Level 0 storage vector pStA0 and subsequent use in Start-Stop Assembly. They can be added by PCR using primer tails (Table S2) or included in the design of synthetic DNA sequences. The prefix and suffix sequences include inward-facing BsaI recognition sites (grey box) and corresponding storage fusion sites (F and R; **bold**) for cloning parts into Level 0 storage vector pStA0 using a Level 0 assembly reaction, as well as inward-facing SapI recognition sites (grey box) with corresponding donor fusion sites (α, β, γ, δ or ε depending on the type of part; **bold**) for subsequent multi-part assembly of expression units in Level 1 assembly.

| Part | Prefix | | Suffix |
|---|---|---|---|
| Promoter | GGTCTCA**TGTG**GCTCTTCG**CAG**<br>BsaI F SapI α | -Promoter- | **CCA**TGAAGAGC**GACC**AGAGACC<br>β SapI R BsaI |
| RBS | GGTCTCA**TGTG**GCTCTTCG**CCA**<br>BsaI F SapI β | -RBS- | **ATG**TGAAGAGC**GACC**AGAGACC<br>γ SapI R BsaI |
| CDS | GGTCTCA**TGTG**GCTCTTCG**ATG**<br>BsaI F SapI γ | -CDS- | **TAA**TGAAGAGC**GACC**AGAGACC<br>δ SapI R BsaI |
| Terminator | GGTCTCA**TGTG**GCTCTTCG**TAA**<br>BsaI F SapI δ | -Terminator- | **GGA**TGAAGAGC**GACC**AGAGACC<br>ε SapI R BsaI |

**Table S2. PCR primer tails for amplification of genetic parts to be cloned in Level 0 vector pStA0 using a Level 0 assembly reaction.** These primer tails contain the prefix and suffix sequences described in Table S1, which include inward-facing BsaI recognition sites (grey box) and corresponding storage fusion sites (F and R; **bold**) for cloning parts into Level 0 storage vector pStA0 using a Level 0 assembly reaction, as well as inward-facing SapI recognition sites (grey box) with corresponding donor fusion sites (α, β, γ, δ or ε depending on the type of part; **bold**) for subsequent multi-part assembly of expression units in Level 1 assembly. In order to improve restriction digestion, additional nucleotides are included at the 5' end of each primer tail so that the BsaI site will located away from the end of the resultant PCR product.

| Primer tail | Sequence (5′-3′) of primer tail |
| --- | --- |
| Promoter Fw primer tail | AAGGGGTTGGTCTCA**TGTG**GCTCTTCG**CAG**…Annealing region<br>BsaI F SapI α |
| Promoter Rv primer tail | CAGTGTTGGGTCTCT**GGTC**GCTCTTCA**TGG**…Annealing region<br>BsaI R SapI β |
| RBS Fw primer tail | AAGGGGTTGGTCTCA**TGTG**GCTCTTCG**CCA**…Annealing region<br>BsaI F SapI β |
| RBS Rv primer tail | CAGTGTTGGGTCTCT**GGTC**GCTCTTCA**CAT**…Annealing region<br>BsaI R SapI γ |
| CDS Fw primer tail | AAGGGGTTGGTCTCA**TGTG**GCTCTTCG**ATG**…Annealing region<br>BsaI F SapI γ |
| CDS Rv primer tail | CAGTGTTGGGTCTCT**GGTC**GCTCTTCA**TTA**…Annealing region<br>BsaI R SapI δ |
| Terminator Fw primer tail | AAGGGGTTGGTCTCA**TGTG**GCTCTTCG**TAA**…Annealing region<br>BsaI F SapI δ |
| Terminator Rv primer tail | CAGTGTTGGGTCTCT**GGTC**GCTCTTCA**TCC**…Annealing region<br>BsaI R SapI ε |

**Table S3. Genetic parts stored in Level 0 vector pStA0 and other plasmids used in this study.**

| Plasmid Name | Accession Number | Comments (Part, selection marker(s), replicon, ID number) |
|---|---|---|
| pStA0::J23100 | MG649435 | Promoter P1 = J23100, $Amp^R$, pMB1, ID = pGT323 |
| pStA0::J23102 | MG649436 | Promoter P2 = J23102, $Amp^R$, pMB1, ID = pGT324 |
| pStA0::J23107 | MG649437 | Promoter P4 = J23107, $Amp^R$, pMB1, ID = pGT326 |
| pStA0::J23113 | MG649438 | Promoter P6 = J23113, $Amp^R$, pMB1, ID = pGT328 |
| pStA0::J23116 | MG649439 | Promoter P5 = J23116, $Amp^R$, pMB1, ID = pGT327 |
| pStA0::J23118 | MG649440 | Promoter P3 = J23118, $Amp^R$, pMB1, ID = pGT336 |
| pStA0::RBSc13 | MG649441 | RBS R3 = RBSc13, $Amp^R$, pMB1, ID = pGT330 |
| pStA0::RBSc33 | MG649442 | RBS R2 = RBSc33, $Amp^R$, pMB1, ID = pGT331 |
| pStA0::RBSc44 | MG649445 | RBS R1 = RBSc44, $Amp^R$, pMB1, ID = pGT332 |
| pStA0::RBSc58 | MG649446 | RBS R4 = RBSc58, $Amp^R$, pMB1, ID = pGT333 |
| pStA0::RBSc36 | MG649443 | RBS R6 = RBSc36, $Amp^R$, pMB1, ID = pGT334 |
| pStA0::RBSc42 | MG649444 | RBS R5 = RBSc42, $Amp^R$, pMB1, ID = pGT335 |
| pStA0::L3S2P55 | MG649450 | Terminator T1 = L3S2P55, $Amp^R$, pMB1, ID = pGT337 |
| pStA0::L3S2P21 | MG649449 | Terminator T2 = L3S2P21, $Amp^R$, pMB1, ID = pGT338 |
| pStA0::ECK120033737 | MG649448 | Terminator T3 = ECK120033737, $Amp^R$, pMB1, ID = pGT339 |
| pStA0::ECK120019600 | MG649447 | Terminator T4 = ECK120019600, $Amp^R$, pMB1, ID = pGT340 |
| pStA0::*crtI* | - | *crtI* CDS, $Amp^R$, pMB1, ID = pGT356 |
| pStA0::*crt*B | - | *crtB* CDS, $Amp^R$, pMB1, ID = pGT357 |
| pStA0::*crtE* | - | *crtE* CDS, $Amp^R$, pMB1, ID = pGT358 |
| pStA0::*dxs* | - | *dxs* CDS, $Amp^R$, pMB1, ID = pGT359 |
| pStA0::*crtW* | - | *crtW* CDS, $Amp^R$, pMB1, ID = pGT360 |
| pStA0::*crtZ* | - | *crtZ* CDS, $Amp^R$, pMB1, ID =pGT361 |
| pStA0::*lcyB* | - | *lcyB* CDS, $Amp^R$, pMB1, ID = pGT362 |
| pStA0::*idi* | - | *idi* CDS, $Amp^R$, pMB1, ID = pGT363 |
| pStA0::*eyfp* | - | *efyp* CDS, $Amp^R$, pMB1, ID = pGT431 |
| **Plasmid Name** | **Accession Number** | **Comments (Selection marker(s), replicon, comments)** |
| pCK302 | KU555410 | $Amp^R$, pBR322 (1) |
| pGT274 | - | $Amp^R$, pBR322, pCK302 with 'No scar' |
| pGT275 | - | $Amp^R$, pBR322, pCK302 with BioBrick scar |
| pGT276 | - | $Amp^R$, pBR322, pCK302 with MoClo scar |
| pGT277 | - | $Amp^R$, pBR322, pCK302 with BASIC scar |

| | | |
|---|---|---|
| pUC19 | M77789 | $Amp^R$, pMB1 (7) |
| pACYC184 | X06403 | $Cam^R$, $Tet^R$, p15A (8) |
| pACYC177 | X06402 | $Amp^R$, $Kan^R$, p15A (8) |
| pGT421 | - | $Amp^R$, pMB1, plasmid construction intermediate |
| pGT422 | - | $Tet^R$, p15A, plasmid construction intermediate |
| pGT410 | - | $Kan^R$, p15A, plasmid construction intermediate |
| pGT414 | - | $Cam^R$, p15A, plasmid construction intermediate |
| pGT411 | - | $Kan^R$, p15A, plasmid construction intermediate |
| pGT412 | - | $Kan^R$, p15A, plasmid construction intermediate |
| pGT413 | - | $Kan^R$, p15A, plasmid construction intermediate |

**Table S4. List of oligonucleotides used in this study.** 5'-phosphorylated oligonucleotides denoted /5Phos/. Construction primers are described in Supplementary Materials and Methods.

| Primer | Sequence (5'-3') | Comments |
|---|---|---|
| OligoGT234 | GGGGAAACGCCTGGTATCT | pStA0 Fw sequencing primer |
| OligoGT235 | AGCAAAAACAGGAAGGCAAA | pStA0 Rv sequencing primer |
| OligoGT339 | GTTGAGGACCCGGCTAGG | pStA1 Fw sequencing primer |
| OligoGT340 | TGTGACGGAAGATCACTTCG | pStA1 Rv sequencing primer |
| OligoGT573 | CCTCGGTGAGTTTTCTCCTTC | pStA2 Fw sequencing primer |
| OligoGT486 | GATTACGCGCAGACCAAAAC | pStA2 Rv sequencing primer and pStA3 Fw sequencing primer |
| OligoGT487 | AAACGGTTAGCGCTTCGTTA | pStA3 Rv sequencing primer |
| OligoGT249 | TGGAGTTCTGAGGTCATTACTGGATCTATCAACAGGAGTCCAAGGC GCTCGGTCGTTCGGCT | Construction primer |
| OligoGT250 | AAAACGAAAGGCCCAGTCTTTCGACTGAGCCTTTCGTTTTATTTGA TGCCCGAAAGGGCCTCGTGATACGCC | Construction primer |
| OligoGT251 | CCGGTGAGCGTGGGTCCCGCGGTATCATTGCAGCAC | Construction primer |
| OligoGT252 | GTGCTGCAATGATACCGCGGGACCCACGCTCACCGG | Construction primer |
| OligoGT253 | TTGGTCTCTCACACTGGATTCTCACC | Construction primer |
| OligoGT254 | GGTCTCAGACCTCTAGGGCG | Construction primer |
| OligoGT255 | CTATGCGGCATCAGAGCAGATTG | Construction primer |
| OligoGT256 | GGGCAGTGAGCGCAACGC | Construction primer |
| OligoGT257 | CAAAAGTTGGCCCAGGGCT | Construction primer |
| OligoGT258 | GCTCAGGTCGCAGACGT | Construction primer |
| OligoGT259 | GTTGCATGATAAAGAAAACAGTCATAAGTGC | Construction primer |
| OligoGT260 | CGCACTTATGACTGTTTTCTTTATCATGCAA | Construction primer |
| OligoGT261 | /5Phos/GCCGCAGCCGAACGAC | Construction primer |
| OligoGT261 | /5Phos/GCCGCAGCCGAACGAC | Construction primer |
| OligoGT262 | /5Phos/GCGTATCACGAGGCCCT | Construction primer |
| OligoGT265 | /5Phos/ATTTGTCCTACTCAGGAGAGCGT | Construction primer |
| OligoGT266 | TACTGGCTCTTCTCTGCTCCTGAGACCATTCTCACCAATAAAAAAC GCCCG | Construction primer |
| OligoGT268 | /5Phos/AGAGACCATTTGTCCTACTCAGGAGAG | Construction primer |
| OligoGT270 | TACTGGCTCTTCTCTGCATTTGAGACCATTCTCACCAATAAAAAAC GCC | Construction primer |

| | | |
|---|---|---|
| OligoGT273 | TACTGGCTCTTCTCTGACCTTGAGACCATTCTCACCAATAAAAAACGCC | Construction primer |
| OligoGT276 | TACTGGCTCTTCTCTGAAGCTGAGACCATTCTCACCAATAAAAAACGCC | Construction primer |
| OligoGT279 | /5Phos/TGAGACCATTCTCACCAATAAAAAACGCC | Construction primer |
| OligoGT289 | TACTGGGTCTCTCTCCGGCATTGTCTTCATTCTCACCAATAAAAAACGCCCG | Construction primer |
| OligoGT297 | CTTTATCATGCAACTCGTAGGACAGG | Construction primer |
| OligoGT298 | AATTTAACTGTGATAAACTACCGCATTAAAGCT | Construction primer |
| OligoGT299 | TACTGGAAGACTTGGCAATTCTCACCAATAAAAAACGCCCG | Construction primer |
| OligoGT329 | ACGCTGAAAAGCGTCTTTTTTCGTTTTGGTCCGGACGAAGAGCGACCTCTAGGGCGGCGG | Construction primer |
| OligoGT330 | CTTATTGTTCGTCTTTGGTACCGAGTTATGAAGAGCCACACTGGATTCTCACCAATAAAAAACGC | Construction primer |
| OligoGT331 | CCCGAAAGGGGGGCCTTTTTTCGTTTTGGTCCGGACGAAGAGCGACCTCTAGGGCGGCGG | Construction primer |
| OligoGT332 | AGGCCTCTTTTCTGGAATTTGGTACCGAGTTATGAAGAGCCACACTGGATTCTCACCAATAAAAAACGC | Construction primer |
| OligoGT333 | TGACAGTGCGGGCTTTTTTTTTcgaccaaaggGGACGAAGAGCGACCTCTAGGGCGGCGG | Construction primer |
| OligoGT334 | GGTGCGGGCTTTTTTctgtgtttccTTATGAAGAGCCACACTGGATTCTCACCAATAAAAAACGC | Construction primer |
| OligoGT335 | GTAATGCGGTGGACAGGATCGGCGGTTTTCTTTTCTCTTCTCAAGGACGAAGAGCGACCTCTAGGGCGGCGG | Construction primer |
| OligoGT336 | TGCAAGGTAGTGGACAAGACCGGCGGTCTTAAGTTTTTTGGCTGAATTATGAAGAGCCACACTGGATTCTCACCAATAAAAAACGC | Construction primer |
| OligoGT356 | GGTCTCAGGAGCAGAGAAGAGCCAGTAGGGCAGTGAGCGCAACG | Construction primer |
| OligoGT357 | GGTCTCTAGTATCCTGAAGAGCGGAAACTATGCGGCATCAGAGCAGATTG | Construction primer |
| OligoGT358 | aatGCAGAGAAGAGCCAGTAGGGC | Construction primer |
| OligoGT359 | aggtCAGAGAAGAGCCAGTAGGGC | Construction primer |
| OligoGT360 | gcttCAGAGAAGAGCCAGTAGGGC | Construction primer |
| OligoGT361 | cgctCAGAGAAGAGCCAGTAGGGC | Construction primer |
| OligoGT362 | CATTTCCTGAAGAGCGGAAACTATGCG | Construction primer |
| OligoGT363 | ACCTTCCTGAAGAGCGGAAACTATGCG | Construction primer |
| OligoGT364 | AAGCTCCTGAAGAGCGGAAACTATGCG | Construction primer |
| OligoGT365 | AGCGTCCTGAAGAGCGGAAACTATGCG | Construction primer |
| OligoGT366 | GGTCTCAAATGCAGAGAAGAGCCAGTAGGGCAGTGAGCGCAAC | Construction primer |

| | | |
|---|---|---|
| OligoGT367 | GGTCTCAAGGTCAGAGAAGAGCCAGTAGGGCAGTGAGCGCAAC | Construction primer |
| OligoGT368 | GGTCTCAGCTTCAGAGAAGAGCCAGTAGGGCAGTGAGCGCAAC | Construction primer |
| OligoGT375 | /5Phos/GAAGACAATGCCGGAGAGAGACCCAGTACCAGTAGGGCAGTGAGCGCAAC | Construction primer |
| OligoGT376 | /5Phos/GAAGACTTTAGTAGTATGAGACCGGAAAGGAAACTATGCGGCATCAGAGC | Construction primer |
| OligoGT377 | GAAGACAATGCCGGAGAGAGACCCAGTAGGGCAGTGAGCGCAACGC | Construction primer |
| OligoGT378 | GAAGACTTTAGTAGTATGAGACCGGAAACTATGCGGCATCAGAGCAGATTG | Construction primer |
| OligoGT379 | GAAGACAAACTAGGAGAGAGACCCAGTAGGGCAGTGAGCGCAACGC | Construction primer |
| OligoGT380 | TACTGGGTCTCTCTCCTAGTTTGTCTTCATTCTCACCAATAAAAAACGCCCG | Construction primer |
| OligoGT381 | GAAGACTTGTAAAGTATGAGACCGGAAACTATGCGGCATCAGAGCAGATTG | Construction primer |
| OligoGT382 | GAAGACAATTACGGAGAGAGACCCAGTAGGGCAGTGAGCGCAACGC | Construction primer |
| OligoGT383 | TACTGGGTCTCTCTCCGTAATTGTCTTCATTCTCACCAATAAAAAACGCCCG | Construction primer |
| OligoGT384 | GAAGACTTCTCGAGTATGAGACCGGAAACTATGCGGCATCAGAGCAGATTG | Construction primer |
| OligoGT385 | TGCCAAGTCTTCCAGTAGGGCAGTGAGCGCAACGC | Construction primer |
| OligoGT386 | GTAATTGTCTTCGGAAACTATGCGGCATCAGAGCAGATTG | Construction primer |
| OligoGT387 | CTCGTTGTCTTCGGAAACTATGCGGCATCAGAGCAGATTG | Construction primer |
| OligoGT448 | tacgaccagtctaaaaagcgcc | Construction primer |
| OligoGT463 | NNNNNNNNNNNNNNNAAAGGAGGTNNNNNNNatgcgtaaaggcgaagagctg | Construction primer |
| OligoGT468 | AAAANNNatgcgtaaaggcgaagagctg | Construction primer |
| OligoGT471 | ATAGTCCatgcgtaaaggcgaagagctg | Construction primer |
| OligoGT472 | acctcctaaaagttaaacaaaattatttctagaggg | Construction primer |
| OligoGT482 | AAATCTGGAGCCGGTGAGCGT | Construction primer |
| OligoGT483 | TAATTTCCCCCAAGATTAGAAAAACTCATCGAGCATCAAATG | Construction primer |
| OligoGT503 | /5Phos/CTGCGAAGAGCCACACTGGATTCTCACCAATAAAAAACG | Construction primer |
| OligoGT504 | ttgacggctagctcagtcctaggtacagtgctagcCCATGAAGAGCGTAAGACCTCTAGGGCGGCG | Construction primer |
| OligoGT505 | ttgacagctagctcagtcctaggtactgtgctagcCCATGAAGAGCGTAAGACCTCTAGGGCGGCG | Construction primer |
| OligoGT507 | tttacggctagctcagccctaggtattatgctagcCCATGAAGAGCGTAAGACCTCTAGGGCGGCG | Construction primer |

| | | |
|---|---|---|
| OligoGT508 | ttgacagctagctcagtcctagggactatgctagcCCATGAAGAGC GTAAGACCTCTAGGGCGGCG | Construction primer |
| OligoGT509 | ctgatggctagctcagtcctagggattatgctagcCCATGAAGAGC GTAAGACCTCTAGGGCGGCG | Construction primer |
| OligoGT510 | /5Phos/TGGCGAAGAGCCACACTGGATTCTCACCAATAAAAAACG | Construction primer |
| OligoGT512 | CTACGTTTTTTAGAAAAAGGAGGTATGCGAGATGTGAAGAGCGTAA GACCTCTAGGGCGGCG | Construction primer |
| OligoGT513 | AAAACACTAGACTGGAAAGGAGGTAGAGAATATGTGAAGAGCGTAA GACCTCTAGGGCGGCG | Construction primer |
| OligoGT514 | ATCGGATTGGATCCAAAGGAGGTTATACCGATGTGAAGAGCGTAAG ACCTCTAGGGCGGCG | Construction primer |
| OligoGT515 | CATGATCGAATGATTAAAGGAGGTTGGAGGTATGTGAAGAGCGTAA GACCTCTAGGGCGGCG | Construction primer |
| OligoGT516 | AGCTCCTTAGCTCCTAAAGGAGGTAGTACATATGTGAAGAGCGTAA GACCTCTAGGGCGGCG | Construction primer |
| OligoGT517 | ACAGGATACATCTGTAAAGGAGGTAACGATGATGTGAAGAGCGTAA GACCTCTAGGGCGGCG | Construction primer |
| OligoGT568 | ttgacggctagctcagtcctaggtattgtgctagcCCATGAAGAGC GTAAGACCTCTAGGGCGGCG | Construction primer |
| OligoGT569 | /5Phos/CGGTATCATTGCAGCACTGGGG | Construction primer |
| OligoGT570 | CGCTCACCGGCTCCAGATTTG | Construction primer |
| OligoGT614 | AAGGGGTTGGTCTCATGTGGCTCTTCGATGGTGAGCAAGGGCGAG | Construction primer |
| OligoGT615 | AAGGGGTTGGTCTCTGGTCTTACGCTCTTCATTACTTGTACAGCTC GTCCATGCC | Construction primer |
| OligoGT657 | ATACTAGatgcgtaaaggcgaagagctg | Construction primer |
| OligoGT659 | AAAAAAAatgcgtaaaggcgaagagctg | Construction primer |
| OligoGT660 | /5Phos/acctcctaaaagttaaacaaaattatttctagagg g | Construction primer |

**Table S5. Spacers implemented as double-stranded linkers.** Spacers can be used in either α-β format in place of a promoter, or δ-ε format in place of a terminator. Here we show each of the 16 spacers as double-stranded linkers in both the α-β and δ-ε configurations. Fusion site cohesive ends are shown in **bold**. Spacers are obtained as two single-stranded oligonucleotides and then mixed and annealed together (described in Supplementary Materials and Methods) to generate the spacer as a linker part that can be used directly in Level 1 assembly reactions.

<table>
<tr><th>Spacer</th><th>Spacer sequence in double-stranded linker</th><th>Forward oligonucleotide</th><th>Reverse oligonucleotide</th></tr>
<tr><td>Spacer 1<br>α-β format</td><td>5′-**CAG**TGGTCAGCGACT-3′<br>| | | | | | | | | | | |<br>3′-ACCAGTCGCTGA**GGT**-5′</td><td>oligoGT538:<br>CAGTGGTCAGCGACT</td><td>oligoGT539:<br>TGGAGTCGCTGACCA</td></tr>
<tr><td>Spacer 1<br>δ-ε format</td><td>5′-**TAA**TGGTCAGCGACT-3′<br>| | | | | | | | | | | |<br>3′-ACCAGTCGCTGA**CCT**-5′</td><td>oligoGT540:<br>TAATGGTCAGCGACT</td><td>oligoGT541:<br>TCCAGTCGCTGACCA</td></tr>
<tr><td>Spacer 2<br>α-β format</td><td>5′-**CAG**GCTGCCGTGAAT-3′<br>| | | | | | | | | | | |<br>3′-CGACGGCACTTA**GGT**-5′</td><td>oligoGT542:<br>CAGGCTGCCGTGAAT</td><td>oligoGT543:<br>TGGATTCACGGCAGC</td></tr>
<tr><td>Spacer 2<br>δ-ε format</td><td>5′-**TAA**GCTGCCGTGAAT-3′<br>| | | | | | | | | | | |<br>3′-CGACGGCACTTA**CCT**-5′</td><td>oligoGT544:<br>TAAGCTGCCGTGAAT</td><td>oligoGT545:<br>TCCATTCACGGCAGC</td></tr>
<tr><td>Spacer 3<br>α-β format</td><td>5′-**CAG**GGCACGCTCAAT-3′<br>| | | | | | | | | | | |<br>3′-CCGTGCGAGTTA**GGT**-5′</td><td>oligoGT546:<br>CAGGGCACGCTCAAT</td><td>oligoGT547:<br>TGGATTGAGCGTGCC</td></tr>
<tr><td>Spacer 3<br>δ-ε format</td><td>5′-**TAA**GGCACGCTCAAT-3′<br>| | | | | | | | | | | |<br>3′-CCGTGCGAGTTA**CCT**-5′</td><td>oligoGT548:<br>TAAGGCACGCTCAAT</td><td>oligoGT549:<br>TCCATTGAGCGTGCC</td></tr>
<tr><td>Spacer 4<br>α-β format</td><td>5′-**CAG**AGTCCGTGCTCA-3′<br>| | | | | | | | | | | |<br>3′-TCAGGCACGAGT**GGT**-5′</td><td>oligoGT550:<br>CAGAGTCCGTGCTCA</td><td>oligoGT551:<br>TGGTGAGCACGGACT</td></tr>
<tr><td>Spacer 4<br>δ-ε format</td><td>5′-**TAA**AGTCCGTGCTCA-3′<br>| | | | | | | | | | | |<br>3′-TCAGGCACGAGT**CCT**-5′</td><td>oligoGT552:<br>TAAAGTCCGTGCTCA</td><td>oligoGT553:<br>TCCTGAGCACGGACT</td></tr>
<tr><td>Spacer 5<br>α-β format</td><td>5′-**CAG**ATTCTGTGCCGC-3′<br>| | | | | | | | | | | |<br>3′-TAAGACACGGCG**GGT**-5′</td><td>oligoGT554:<br>CAGATTCTGTGCCGC</td><td>oligoGT555:<br>TGGGCGGCACAGAAT</td></tr>
<tr><td>Spacer 5<br>δ-ε format</td><td>5′-**TAA**ATTCTGTGCCGC-3′<br>| | | | | | | | | | | |<br>3′-TAAGACACGGCG**CCT**-5′</td><td>oligoGT556:<br>TAAATTCTGTGCCGC</td><td>oligoGT557:<br>TCCGCGGCACAGAAT</td></tr>
<tr><td>Spacer 6<br>α-β format</td><td>5′-**CAG**ATCAACGCCTGC-3′<br>| | | | | | | | | | | |<br>3′-TAGTTGCGGACG**GGT**-5′</td><td>oligoGT558:<br>CAGATCAACGCCTGC</td><td>oligoGT559:<br>TGGGCAGGCGTTGAT</td></tr>
<tr><td>Spacer 6<br>δ-ε format</td><td>5′-**TAA**ATCAACGCCTGC-3′<br>| | | | | | | | | | | |<br>3′-TAGTTGCGGACG**CCT**-5′</td><td>oligoGT560:<br>TAAATCAACGCCTGC</td><td>oligoGT561:<br>TCCGCAGGCGTTGAT</td></tr>
</table>

| | | | |
|---|---|---|---|
| Spacer 7<br>α-β format | 5′-**CAG**ATCTGCGGCAAC-3′<br>\|\|\|\|\|\|\|\|\|\|\|\|<br>3′-TAGACGCCGTTG**GGT**-5′ | oligoGT562:<br>CAGATCTGCGGCAAC | oligoGT563:<br>TGGGTTGCCGCAGAT |
| Spacer 7<br>δ-ε format | 5′-**TAA**ATCTGCGGCAAC-3′<br>\|\|\|\|\|\|\|\|\|\|\|\|<br>3′-TAGACGCCGTTG**CCT**-5′ | oligoGT564:<br>TAAATCTGCGGCAAC | oligoGT565:<br>TCCGTTGCCGCAGAT |
| Spacer 8<br>α-β format | 5′-**CAG**TGCGACCTGACT-3′<br>\|\|\|\|\|\|\|\|\|\|\|\|<br>3′-ACGCTGGACTGA**GGT**-5′ | oligoGT566:<br>CAGTGCGACCTGACT | oligoGT567:<br>TGGAGTCAGGTCGCA |
| Spacer 8<br>δ-ε format | 5′-**TAA**TGCGACCTGACT-3′<br>\|\|\|\|\|\|\|\|\|\|\|\|<br>3′-ACGCTGGACTGA**CCT**-5′ | oligoGT616:<br>TAATGCGACCTGACT | oligoGT617:<br>TCCAGTCAGGTCGCA |
| Spacer 9<br>α-β format | 5′-**CAG**AGGTGTCTCGCA-3′<br>\|\|\|\|\|\|\|\|\|\|\|\|<br>3′-TCCACAGAGCGT**GGT**-5′ | oligoGT618:<br>CAGAGGTGTCTCGCA | oligoGT619:<br>TGGTGCGAGACACCT |
| Spacer 9<br>δ-ε format | 5′-**TAA**AGGTGTCTCGCA-3′<br>\|\|\|\|\|\|\|\|\|\|\|\|<br>3′-TCCACAGAGCGT**CCT**-5′ | oligoGT620:<br>TAAAGGTGTCTCGCA | oligoGT621:<br>TCCTGCGAGACACCT |
| Spacer 10<br>α-β format | 5′-**CAG**GCTACAGGCTGC-3′<br>\|\|\|\|\|\|\|\|\|\|\|\|<br>3′-CGATGTCCGACG**GGT**-5′ | oligoGT622:<br>CAGGCTACAGGCTGC | oligoGT623:<br>TGGGCAGCCTGTAGC |
| Spacer 10<br>δ-ε format | 5′-**TAA**GCTACAGGCTGC-3′<br>\|\|\|\|\|\|\|\|\|\|\|\|<br>3′-CGATGTCCGACG**CCT**-5′ | oligoGT624:<br>TAAGCTACAGGCTGC | oligoGT625:<br>TCCGCAGCCTGTAGC |
| Spacer 11<br>α-β format | 5′-**CAG**TCAGACGGCACT-3′<br>\|\|\|\|\|\|\|\|\|\|\|\|<br>3′-AGTCTGCCGTGA**GGT**-5′ | oligoGT626:<br>CAGTCAGACGGCACT | oligoGT627:<br>TGGAGTGCCGTCTGA |
| Spacer 11<br>δ-ε format | 5′-**TAA**TCAGACGGCACT-3′<br>\|\|\|\|\|\|\|\|\|\|\|\|<br>3′-AGTCTGCCGTGA**CCT**-5′ | oligoGT628:<br>TAATCAGACGGCACT | oligoGT629:<br>TCCAGTGCCGTCTGA |
| Spacer 12<br>α-β format | 5′-**CAG**ATCGCAACTGGC-3′<br>\|\|\|\|\|\|\|\|\|\|\|\|<br>3′-TAGCGTTGACCG**GGT**-5′ | oligoGT630:<br>CAGATCGCAACTGGC | oligoGT631:<br>TGGGCCAGTTGCGAT |
| Spacer 12<br>δ-ε format | 5′-**TAA**ATCGCAACTGGC-3′<br>\|\|\|\|\|\|\|\|\|\|\|\|<br>3′-TAGCGTTGACCG**CCT**-5′ | oligoGT632:<br>TAAATCGCAACTGGC | oligoGT633:<br>TCCGCCAGTTGCGAT |
| Spacer 13<br>α-β format | 5′-**CAG**GGCAATCGTGCT-3′<br>\|\|\|\|\|\|\|\|\|\|\|\|<br>3′-CCGTTAGCACGA**GGT**-5′ | oligoGT634:<br>CAGGGCAATCGTGCT | oligoGT635:<br>TGGAGCACGATTGCC |
| Spacer 13<br>δ-ε format | 5′-**TAA**GGCAATCGTGCT-3′<br>\|\|\|\|\|\|\|\|\|\|\|\|<br>3′-CCGTTAGCACGA**CCT**-5′ | oligoGT636:<br>TAAGGCAATCGTGCT | oligoGT637:<br>TCCAGCACGATTGCC |
| Spacer 14<br>α-β format | 5′-**CAG**ATTGCCTGCGTC-3′<br>\|\|\|\|\|\|\|\|\|\|\|\|<br>3′-TAACGGACGCAG**GGT**-5′ | oligoGT638:<br>CAGATTGCCTGCGTC | oligoGT639:<br>TGGGACGCAGGCAAT |
| Spacer 14<br>δ-ε format | 5′-**TAA**ATTGCCTGCGTC-3′<br>\|\|\|\|\|\|\|\|\|\|\|\|<br>3′-TAACGGACGCAG**CCT**-5′ | oligoGT640:<br>TAAATTGCCTGCGTC | oligoGT641:<br>TCCGACGCAGGCAAT |

<table>
<tr><td>Spacer 15<br>α-β format</td><td>5′-CAGGCACCAATCGCT-3′<br>||||||||||||<br>3′-CGTGGTTAGCGAGGT-5′</td><td>oligoGT642:<br>CAGGCACCAATCGCT</td><td>oligoGT643:<br>TGGAGCGATTGGTGC</td></tr>
<tr><td>Spacer 15<br>δ-ε format</td><td>5′-TAAGCACCAATCGCT-3′<br>||||||||||||<br>3′-CGTGGTTAGCGACCT-5′</td><td>oligoGT644:<br>TAAGCACCAATCGCT</td><td>oligoGT645:<br>TCCAGCGATTGGTGC</td></tr>
<tr><td>Spacer 16<br>α-β format</td><td>5′-CAGAGCAATCCACGC-3′<br>||||||||||||<br>3′-TCGTTAGGTGCGGGT-5′</td><td>oligoGT646:<br>CAGAGCAATCCACGC</td><td>oligoGT647:<br>TGGGCGTGGATTGCT</td></tr>
<tr><td>Spacer 16<br>δ-ε format</td><td>5′-TAAAGCAATCCACGC-3′<br>||||||||||||<br>3′-TCGTTAGGTGCGCCT-5′</td><td>oligoGT648:<br>TAAAGCAATCCACGC</td><td>oligoGT649:<br>TCCGCGTGGATTGCT</td></tr>
</table>

**Table S6. List of synthetic DNA sequences used in the study**

| Synthetic DNA sequence name | Sequence |
|---|---|
| *crtI* recoded (1647 bp) | atgacatcagctctcccccgcccggcaccaagtccgtacgcacgccgtaaaacggcgttggttatt<br>ggcgcaggttttggtgggttggccctgggcattcgtctacagtcgttaggttttgatacaacaatt<br>ttggaacgtctggatggtcctggtggtcgcgcgtatcaaaaacgtaccccagatggctatgtcttt<br>gacatgggtccgactgtgctgacggtgccgcattttatcgaagaactgtttgcgcttgaacgtgat<br>cgtgccggcctggatgcccccgattatcctcctgaagtgttgtcgggcgagcgcgttaaggaaggc<br>gtttctggtggcccgcatacgagccggtatgtcaccttagtgccgattctgcccttttaccgcatt<br>gtttttcacgatggcacgtattttgattatgatggcgaccctgaaagtactcggcggcagattgct<br>gaattggcccctggcgacttagccgggtatgaacgctttcatgccgatgccgaggccatctttcgt<br>cggggcttcctggaactgggctacacgcactttggtgacgtcccgacgatgctgcgggttgtacct<br>gatctgctcaaactggacgccgttcggaccctgttctcctttacgagtaagtactttcagagcgac<br>aaactgcgccaagtgttctcttttgaaacccttttggtgggtgggaatcctctgagtgtgccggcg<br>atctatgcaatgattcacttcgttgaaaagacttgggggatccactatgccatgggcggcacaggc<br>gcactggtgcgcggcctagtccaaaaatttgaggagctgggtggcgccattcgttatggcgccggc<br>gtcgatgaagtactggtggatggcaatctgcctggtaaacgcacagcgcggggtgtgcgcctggaa<br>agcggcgaagaactgcgcgccgacctggtggcgtccaatggcgattgggctaacacgtatctgaaa<br>cgcgtccggccatcggcacgtctggtcaactctgatttacgcgtgaaagccgcatctgaaagtatg<br>agcctgctcgtggtttatttcgggtttcgcggcggtgatgacctgcccctcaaacatcataatatt<br>ttattaggcccacgctacgaggctctgctgagcgaaatctttggcacaaaacggttgggcgaagat<br>tttagccagtacctgcacgtcccaacgctcaccgatccggctctggcacccgcgggtcatcatgcg<br>gcctatacacttgtcccggtgccgcataatggctcgggtattgattgggacgtggaaggtccaaag<br>cttgccgaagcagccctggcagatatcgagcgccgcggtttgattccgggcctccgtgaacggctc<br>acacattttgaatttattacgccagattatttcgcaggcactctcgattcctatctggggaacgcg<br>tttggtccggagccgcgtctggtccagtcggcatttttccgcccgcacaaccgcagcgaggatctc<br>cacaacttttacttagtcggggcgggcgcgcagccaggcgcaggcacaccgagcgttatgatgtcc<br>gcgaaaatgacagcgcgcctaatcgctgaagatttcggtatccatgctgatatccggcgctaa |
| *crtB* recoded (978 bp) | Atgcgtagtcgcgctggtctgagcttacggttacccacgcgtaccttgaccgtgaccgattactcc<br>cccgccctgccctgcaccgaactgcgccgtcctccactggctcaggcggttcgctactgtcgggat<br>ttgacccgccagcactcaaagaccttctatctgggttcacagctcttttcgcctccggaacgcgcg<br>gcagtttgggcagtgtatgcggcgtgccgcgcgggcgatgacatcgtcgatgaagccggcaacggc<br>gaccgcgaacgcgaattgcgggaatggcgcagccgtattgatgccgcgtttgctggccaaccagcg<br>gatgatcccatctcaaccgcgctggcctgggcggcaggtcggtacgccatcccgcactcagctttc<br>gcggaactgcatgaaggcctcaacatggatttacgcggtcatgaataccgtgatatggatgacttg<br>ttactgtattgccgccgtgtggcaggtgtggttggctttatggtggcaccgatttctggctaccgt<br>ggggggctgctaccctgaatgatgctctccaactagggcaggcgatgcaactgacgaatattctg<br>cgcgatgtcggtgaagatctgacccgcggccgcgtatacctgccacagtctctgcttgatgaatat<br>ggcctgtctcgcgccgcgttagagcgctggggtcagggtgagcccctgtcaccggcctaccgtgct<br>ctcatgactcatcttggcggccttgcacgtgaatggtatgcagcaggtcgtgctggtattcctcaa<br>cttgatggacgcggtcctctcgccgttctgactgccgcccgtgcgtatgagggtattctggacgat<br>ttggaacgggccggctacgacaacttcggtcggcgcgcgtacgtgtcaggtcgtcgtaaacttctg<br>atgttaccgcaggcctggtgggaactgcgtagtctgggcgctgtccacggctaa |
| *crtE* recoded (990 bp) | atgcgcccggaattactcgcacgcgtgttaagcctgttaccggaaacctccgcgacgccggaattg<br>gcacgcttttacgcgctcctgcgcgactatcctcaacgtggtggcaagggcattcggtcagaatta<br>ctgcttgcctctgctcgtgcgcacggcctgtccgagtcagataccggttgggagtcagcattatgg<br>ctggcggcagccttagaactgtttcagaactgggtgctggtgcacgatgatattgaagatgattcg |

gaagaacgccgtggtcgtccggccctgcaccacttgtgtggtatgccggtcgctcttaacgtgggg
gacgcgctgcacgcttacatgtgggctgctgttgggaaagccaatgttccgggagcgtttgaagag
tttctgcagatggtgtaccgcacggcggaaggccagcatctggatctggcatgggtggagggtcgt
gaatggggcctgcgtcccgcggattatctccagatggttggcctgaaaaccgcacactacacggtt
atcgtgccgttacgtctgggggccctggcggcaggcatggcaccgcaggacgcgttcaccccagcg
ggtctggcgctgggtaccgcgtttcagattcgtgacgatgtcctcaatctggcaggtgatccggtg
aagtatggtaaagaaattggtggcgatctgttggaaggtaaacgtactctgattgtcctggactgg
ttgactacggcgccggatgatcgcaaagccatcttcctggaccagatgcgtcggcaccgcgcagat
aaagaccctgcggtgatcgatgaaattcaccgctggctgcttgaaagcggctctgtggaagcggcg
caggactacgcgcaggcacaagccgcggaaggtctggacttgcttgaaaaagcattggcagacgcg
ccggatgcccaggccgccgctgccttactcgcttctgttcgggaactggccacccgcgaaaaataa

*dxs* recoded (1863 bp)

atgtcttttgatattgcgaaatatccaaccctggccctagttgactcgactcaggaattacgcctg
ctgccgaaagagagccttccaaagctgtgcgatgagttacgccgctacctcctggattctgttagt
cgtagctccggccacttcgcatcgggactaggtaccgtcgaactgacggttgcactgcattatgtg
tataacaccccgttcgatcaattaatttgggacgtcggtcatcaagcatatccgcataaaattctg
accggtcgtcgcgacaagatcggcacaattcgtcaaaaaggtggattgcatcctttcccgtggcgc
ggcgagtccgaatacgatgtgttgagcgttggccattcgtcaacttctatcagcgctggtattggt
attgcggtcgccgcggagaaagaaggcaaaaatcggcgtacggtttgcgtgatcggggatggtgca
atcaccgcaggcatggcattcgaagcgatgaaccatgcgggggacattcgtccggatatgctagtg
attctgaacgataacgaaatgagtatttccgagaacgtgggggctcttaataaccacttagcgcag
ctgctgagcggtaaactttattctagcctgcgcgaaggcggtaagaaagtgttctcaggcgtccct
ccgattaaagagctccttaagcgtactgaggaacacattaagggtatggtggttccaggcaccctg
ttcgaagaactgggttttaattacattggtcctgtggacggccatgacgtgttaggcttaattacc
acgttaaaaaacatgcgcgatctgaaaggacctcagtttctccacatcatgaccaagaaaggtcgc
ggctatgaaccggccgagaaagatccgattaccttccacgcagttccgaaattcgacccatcctcc
ggctgtctgccgaaaagcagcggaggcctgccgagctattccaaaatctttggtgactggctgtgc
gaaactgcggcaaaagataacaaactgatggccatcaccccggcaatgcgcgaaggttcgggaatg
gtggaattctctcgcaagttcccagaccgttattttgacgttgctatcgccgagcaacatgcagta
acctttgcggctggtctggcaattggtggttataagccaattgtggccatctattcaacgttctta
caacgtgcatatgaccaggtgctacatgatgtggccattcagaaactgccggtcttatttgccatc
gatcgtgcgggggatcgttggggcagatggtcagacgcatcagggcgccttcgacctaagttatctg
cgctgtatcccagaaatggtgattatgaccccctagcgatgagaatgaatgtcgtcagatgttatac
accggctatcattacaacgacggaccatccgctgtgcgctacccgcgcggaaacgcggtgggcgtt
gaactgaccccgttagaaaaattaccgatcggaaaagggattgttaaacgtcgcggagaaaaactc
gctattctgaacttcggcacacttatgcccgaagcggctaaagtagcggaaagcctaaacgcaacc
ttggtggatatgcgcttcgtaaagccactggacgaagccctaatcttagagatggccgcgtcacat
gaggcgttagttacggtagaggaaaacgctattatgggcggagcgggtagtggtgtgaacgaagtt
ctgatggctcaccgcaaaccggtgccggtactgaacatcggcctcccggatttttcattccccaa
ggtactcaagaggaaatgcgcgccgagctcggcttagatgctgcaggaatggaagccaaaattaag
gcctggcttgcataa

*crtW* recoded (777 bp)

Atggtccagtgccagccgtcgtcgcttcattctgaaaaactcgttcttctctcgagcactattcgc
gatgataaaaacatcaacaagggcattttcatcgcctgctttattttgtttctctggccatttct
ctgattcttctgctgtccattgatacttcaattattcataaatctctgctgggtatcgcaatgctt
tggcagacatttttatataccggcctcttcattacagcgcacgatgctatgcatggcgttgtttat
ccgaaaaacccgcgcattaataatttcattggaaaactgacgctgattctgtatggtctgttaccg
tataaagatctgttaaaaaagcattggcttcatcatggtcatccgggtaccgatctggatccggat
tactacaatggccatccccagaatttttcttatggtacttgcactttatgaaaagctattggcgg
tggacccaaattttcggccttgtgatgattttccacggactgaaaaatctggtacatattccggag
aataatttgatcatcttttggatgattccgtccattttgtccagtgtgcaactcttttactttggg
acattcttaccgcataaaaaaactggaaggcggctataccaatccccactgcgctcgctccattccg
ctcccgctgttctggagctttgtgacatgttatcacttcggctatcataaagaacatcacgaatat
cctcaactcccgtggtggaaactgcctgaagcacacaaaatctcgctgtaa

*crtZ*
recoded
(528 bp)

atgctgtggatttggaatgctctgatcgtgtttgttaccgtcattggcatggaagttatcgcggct
ctggcccacaaatatattatgcatggttggggatggggttggcatttgtcgcaccatgagccgcgc
aagggagcgtttgaagtgaacgatctgtatgcagtagtttttgcggcccttagcattcttctgatt
tatctgggctcgaccggcatgtggcccttgcaatggatcggtgcgggcatgacggcttacggcttg
ctttattttatggttcacgatggattagttcaccagcgctggccattccgttatatcccacgcaaa
ggctatttaaaacgtctttacatggcacatcgtatgcatcatgcggttcggggaaagaaggctgc
gtgagctttgggtttctctatgcgccgcctctttctaaactgcaagctaccctgcgtgaacggcat
ggcgcgcgcgcaggcgcggctcgtgatgcgcagggtggcgaagatgaacccgcatctggcaaataa

*lcyB*
recoded
(1503 bp)

atggatacgcttctgaaaacgcctaataaccttgagtttctgaatccccatcatggttttgcagtg
aaagcgagcaccttccgttccgagaaacatcataatttcgggagtcgcaaattctgtgaaacactt
ggccgctcggtgtgtgtgaagggctcatcgtcggccttattggaacttgtgccggagactaaaaaa
gaaaacttggattttgaactgcccatgtatgacccttctaaaggagtggtggttgatcttgccgta
gtaggagggggcccggcgggcctggccgtagcccagcaggtgagcgaagctggtctgagcgtctgc
tctattgacccgaatccaaaactgatctggccaaacaactacggcgtatgggtggatgagtttgag
gcgatggatctgctggattgcttagatgcgacctggtcgggcgctgcagtgtatattgatgacaat
actgcaaaagaccttcaccgcccttacggtcgtgttaaccgtaaacagttaaaatccaaaatgatg
cagaaatgtatcatgaacggagtcaaattccaccaggcaaaagttattaaagtgattcacgaagaa
agcaaaagcatgctgatttgtaatgacggcatcaccattcaggcgactgtcgtattagatgcgaca
ggctttagccgctctcttgtacaatatgataagccatataacccaggctatcaagttgcatatggc
atccttgcagaggtggaagaacacccatttgacgttaataaaatggtttttatggattggcgtgac
agccacttaaagaacaataccgatctgaaagagcgtaatagtcgcattccgactttcctctatgcc
atgccgtttagtagcaaccgcatttttctggaggaaacaagcctggtcgcccgtccgggccttcgc
atcgatgacattcaggaacgcatggtggcccgcctgaaccatctgggcatcaaagtcaaaagtatt
gaagaagatgaacactgcctgattccgatgggcggtcccctgccggtgcttccgcaacgcgtcgtg
ggcattggcgggactgcgggcatggttcacccttcgaccggatatatggtggcgcggacccttgct
gccgcccctgtggtggctaacgctattattcaatacctgggcagtgaacgtagtcactccggtaat
gaactgtcaactgcagtctggaaggatctgtggccaattgagcgccgtcgccagcgtgaattttt
tgcttcggtatggatattctgctgaaactggatctgccggctacccgtcgcttcttcgatgctttt
ttcgatcttgagccccgctattggcatggctttctgtccagccgtttattcctgccggaactcatc
gttttcggtttgtccttgttttcacatgccagcaacacttctcgttttgaaatcatgacgaaaggg
acggtacctctggttaatatgattaacaacttgttgcaagataaagaataa

*idi*
recoded
(918 bp)

atgctgcgctcgcttctgcgcggtttaactcacatcccgcgcgtcaactcggcccaacagccttca
tgcgcacacgcgcgcttacaatttaagctgcgttcgatgcagatgactctgatgcaaccgagtatc
tcagctaacctgtcgcgcgccgaagatcgtactgaccacatgcgcggtgcaagtacgtgggctggt
ggtcaatcccaagatgaactgatgttaaaggacgaatgcattttggttgatgttgaagataacatt
actggacatgcatcaaagttggaatgtcataaatttctgccgcaccaacccgcaggtttgttgcac
cgcgccttcagtgtatttctgtttgatgaccaaggtcgtctgttgcttcaacaacgggcacgctcg
aaaattacctttccgagtgtatggaccaatacttgttgcagtcatccattgcacggtcagacgcca
gatgaagtggatcaactgagccaggtcgcggacgggaccgtcccgggggctaaggcggccgccatc
cggaaactggaacatgaactgggtatcccggcccatcaactgccagcttcagcgtttcgtttcctc
acccgtttgcattactgtgcggctgacgtgcagccggcggctacgcagtcagcactttgggggaa
catgaaatggattatatcctgttcattcgtgcgaatgtaacactcgccccgaatcctgacgaagtg
gacgaggtccgttatgtaacgcaggaagaactgcgccagatgatgcaaccggataatggccttcag
tggagtccatggttccgtattattgccgcccggtttcttgaacgttggtgggccgatctggacgca
gcactgaacacggataaacatgaagattggggtaccgtgcatcatattaatgaggcataa

**Table S7. Primers used for cloning parts into Level 0 vector pStA0 by inverse PCR**

| Plasmid | Fw primer | Rv primer |
|---|---|---|
| pStA0::J23100 | oligoGT504 | oligoGT503 |
| pStA0::J23102 | oligoGT505 | oligoGT503 |
| pStA0::J23118 | oligoGT568 | oligoGT503 |
| pStA0::J23107 | oligoGT507 | oligoGT503 |
| pStA0::J23116 | oligoGT508 | oligoGT503 |
| pStA0::J23113 | oligoGT509 | oligoGT503 |
| pStA0::RBSc13 | oligoGT510 | oligoGT512 |
| pStA0::RBSc33 | oligoGT510 | oligoGT513 |
| pStA0::RBSc44 | oligoGT510 | oligoGT514 |
| pStA0::RBSc58 | oligoGT510 | oligoGT515 |
| pStA0::RBSc36 | oligoGT510 | oligoGT516 |
| pStA0::RBSc42 | oligoGT510 | oligoGT517 |
| pStA0::L3S2P55 | oligoGT329 | oligoGT330 |
| pStA0::L3S2P21 | oligoGT331 | oligoGT332 |
| pStA0::ECK120033737 | oligoGT333 | oligoGT334 |
| pStA0::ECK120019600 | oligoGT335 | oligoGT336 |

**Table S8. Primer designs to introduce suitable parts into Level 0 vector pStA0 using inverse PCR.** Annealing regions shown (underlined) do not include the *lacZα* gene and BsaI recognition sites, so these are removed from the resultant plasmid. The partial tails shown include SapI recognition sites (grey box) with corresponding donor fusion sites (α, β, γ, δ or ε depending on the type of part; **bold**) for subsequent multi-part assembly of expression units in Level 1 assembly. The sequence of the part being cloned can be added to the tail outside of the fusion sites where indicated. The part sequence can be added to one primer or split between both to avoid excessively long primers. The PCR product is circularised by ligation to form the complete plasmid. Ligation requires a 5' phosphate group to be present at one or both ends of the PCR product, which can be achieved using a 5'-phosphorylated primer as shown (denoted /5Phos/) or by enzymatic phosphorylation of the PCR product, for example using the NEB site-directed mutagenesis kit.

| Primer | Sequence (5′-3′) of primer annealing region and partial tail |
|---|---|
| Promoter forward primer | /5Phos/...Part Sequence...**CCA**TGAAGAGCGACCTCTAGGGCGGCG<br>β SapI Annealing region |
| Promoter reverse primer | Part Sequence...**CTG**CGAAGAGCCACACTGGATTCTCACCAATAAAAAACG<br>α SapI Annealing region |
| RBS forward primer | /5Phos/...Part Sequence...**ATG**TGAAGAGCGACCTCTAGGGCGGCG<br>γ SapI Annealing region |
| RBS reverse primer | Part Sequence...**TGG**CGAAGAGCCACACTGGATTCTCACCAATAAAAAACG<br>β SapI Annealing region |
| CDS forward primer | /5Phos/...Part Sequence...**TAA**TGAAGAGCGACCTCTAGGGCGGCG<br>δ SapI Annealing region |
| CDS reverse primer | Part Sequence...**CAT**CGAAGAGCCACACTGGATTCTCACCAATAAAAAACG<br>γ SapI Annealing region |
| Terminator forward primer | /5Phos/...Part Sequence...**GGA**TGAAGAGCGACCTCTAGGGCGGCG<br>ε SapI Annealing region |
| Terminator reverse primer | Part Sequence...**TTA**CGAAGAGCCACACTGGATTCTCACCAATAAAAAACG<br>δ SapI Annealing region |

## SUPPLEMENTARY REFERENCES


1. Kelly,C.L., Liu,Z., Yoshihara,A., Jenkinson,S.F., Wormald,M.R., Otero,J., Estévez,A., Kato,A., Marqvorsen,M.H.S., Fleet,G.W.J., *et al.* (2016) Synthetic Chemical Inducers and Genetic Decoupling Enable Orthogonal Control of the rhaBAD Promoter. *ACS Synth. Biol.*, **5**, 1136–1145.

2. Kim,D., Hong,J.S.-J., Qiu,Y., Nagarajan,H., Seo,J.-H., Cho,B.-K., Tsai,S.-F. and Palsson,B.Ø. (2012) Comparative analysis of regulatory elements between Escherichia coli and Klebsiella pneumoniae by genome-wide transcription start site profiling. *PLoS Genet.*, **8**, e1002867.

3. Crooks,G.E., Hon,G., Chandonia,J.-M. and Brenner,S.E. (2004) WebLogo: a sequence logo generator. *Genome Res.*, **14**, 1188–1190.

4. Anderson,J.C. (2009) *Registry of Standard Biological Parts*.

5. Bryksin,A.V. and Matsumura,I. (2010) Overlap extension PCR cloning: a simple and reliable way to create recombinant plasmids. *Biotechniques*, **48**, 463–465.

6. Kelly,C.L., Taylor,G.M., Hitchcock,A., Torres-Méndez,A. and Heap,J.T. (2018) A Rhamnose-Inducible System for Precise and Temporal Control of Gene Expression in Cyanobacteria. *ACS Synth. Biol.*, 10.1021/acssynbio.7b00435.

7. Yanisch-Perron,C., Vieira,J. and Messing,J. (1985) Improved M13 phage cloning vectors and host strains: nucleotide sequences of the M13mp18 and pUC19 vectors. *Gene*, **33**, 103–119.

8. Chang,A.C. and Cohen,S.N. (1978) Construction and characterization of amplifiable multicopy DNA cloning vehicles derived from the P15A cryptic miniplasmid. *J. Bacteriol.*, **134**, 1141–1156.
